# Microfluidic-assisted fiber production: Potentials, limitations, and prospects

**DOI:** 10.1063/5.0129108

**Published:** 2022-11-17

**Authors:** Afshin Abrishamkar, Azadeh Nilghaz, Maryam Saadatmand, Mohammadreza Naeimirad, Andrew J. deMello

**Affiliations:** 1 Department of Chemical Engineering, Faculty of Engineering, McMaster University, Hamilton, Ontario L8S 4L8, Canada; 2 Laboratory of Biochemistry, UMR CNRS 8231, Institute of Chemistry Biology Innovation (CBI), ESPCI Paris, PSL University, 10 rue Vauquelin, 75005 Paris, France; 3 Institute for Frontier Materials, Deakin University, Waurn Ponds, Victoria 3216, Australia; 4 Department of Chemical and Petroleum Engineering, Sharif University of Technology, 11155-9465 Tehran, Iran; 5 Department of Materials and Textile Engineering, Faculty of Engineering, Razi University, 67144-14971 Kermanshah, Iran; 6 Department of Chemistry and Applied Biosciences, Institute for Chemical and Bioengineering, ETH Zurich, Vladimir-Prelog-Weg1, 8049 Zurich, Switzerland

## Abstract

Besides the conventional fiber production methods, microfluidics has emerged as a promising approach for the engineered spinning of fibrous materials and offers excellent potential for fiber manufacturing in a controlled and straightforward manner. This method facilitates low-speed prototype synthesis of fibers for diverse applications while providing superior control over reaction conditions, efficient use of precursor solutions, reagent mixing, and process parameters. This article reviews recent advances in microfluidic technology for the fabrication of fibrous materials with different morphologies and a variety of properties aimed at various applications. First, the basic principles, as well as the latest developments and achievements of microfluidic-based techniques for fiber production, are introduced. Specifically, microfluidic platforms made of glass, polymers, and/or metals, including but not limited to microfluidic chips, capillary-based devices, and three-dimensional printed devices are summarized. Then, fiber production from various materials, such as alginate, gelatin, silk, collagen, and chitosan, using different microfluidic platforms with a broad range of cross-linking agents and mechanisms is described. Therefore, microfluidic spun fibers with diverse diameters ranging from submicrometer scales to hundreds of micrometers and structures, such as cylindrical, hollow, grooved, flat, core–shell, heterogeneous, helical, and peapod-like morphologies, with tunable sizes and mechanical properties are discussed in detail. Subsequently, the practical applications of microfluidic spun fibers are highlighted in sensors for biomedical or optical purposes, scaffolds for culture or encapsulation of cells in tissue engineering, and drug delivery. Finally, different limitations and challenges of the current microfluidic technologies, as well as the future perspectives and concluding remarks, are presented.

## I. INTRODUCTION

Fibers are well-known materials with a super-high aspect ratio (i.e., length/diameter) that play an essential role in human life.^1^ Nowadays, fibrous materials, such as nylon filaments, drawn polyester yarns, biodegradable fibers, and carbon and glass roving, have vast applications, respectively, in the textile, automation, medicine, and aerospace industries. Man-made fibers can be produced using polymeric materials through different spinning methods, e.g., melt-spinning, wet-spinning, and dry-spinning in macro-scale processes.^2,3^ Also, electrospinning is the most applicable method for producing fibers at the nanoscale.^4,5^ Among the existing spinning methods, melt-spinning is the most commercial and desirable method for fiber production.^6^ In this technique, molten polymers are typically passed through a spinning orifice, where they are solidified using quench air and drawn to get acceptable mechanical performance before being either winded in filament form using high-speed winders or cut in staple fibers form. The inherent properties of polymers (e.g., thermoplasticity) are a key factor in spinning methods. Thermoset polymers should be dissolved in a solvent, spun into a fiber shape through two solution-spinning methods, and solidified by either solvent coagulation using a non-solvent (i.e., wet-spinning) or evaporation via hot air (i.e., dry-spinning).^3^

Spun fibers may consist of one, two, or more polymers in separate or blend forms, known as mono-component, bi-component, or multi-component fibers, respectively. Bi-component fibers are divided into four groups: side-by-side, core–shell, segmented-pies, and islands-in-the-sea.^2^ In terms of geometry, fibers can have different cross sections ranging from round, ribbon, and hollow to delta and trilobal, as well as other profiles depending on the shape of the spinning orifice geometry. Microfibers are an appealing class of products in textile technology due to their high surface area and low linear density (i.e., less than one denier: 1 g per 9000 m).^7^ Besides the aforementioned conventional techniques, the fabrication of fibrous structures and microfibers has been significantly advanced by taking advantage of microfluidic-assisted technologies.^8^ Microfluidic technologies enable the fabrication of fibers with a variety of properties targeting various applications while providing superior control over reaction conditions, efficient use of precursor solutions, mixing of the reagents, and process conditions.^9–12^ In addition, these approaches offer a wide range of possibilities in pre-, on-site-, and post-fabrication treatments, allowing fabrication, manipulation, and processing of fibers; all made possible using a single setup.^13,14^

From a technological viewpoint, fluid behavior on the microscopic scale can be remarkably different from what is observed on the macro-scale, allowing atypical and unintuitive phenomena to be commonplace.^15–18^ As a well-known branch of science and technology, microfluidics has been at the forefront of technologies, paving the way for the miniaturization of experimental platforms for different applications.^19–21^ Indeed, the field of microfluidics has experienced tremendous development during the past two decades, mainly through dealing with the investigation of fluids and their behaviors in submillimeter dimensions.^22–24^ In addition, the manipulation of these fluid behaviors has gained considerable attention in different scientific communities to favor a variety of topics, e.g., cell culture,^25^ diagnostics,^26^ micro-encapsulation,^27^ tissue engineering,^28^ and fiber production.^29^ Microfluidic is an interdisciplinary technology that deals with the manipulation and processing of small fluid volumes using microscale channel structures, thus offering pronounced advantages for the production of fiber-based structures, including the minimal consumption of materials, short reaction time for the formation of structures, low fabrication cost, high synthesis yields, and simple apparatus and operation.

**FIG. 1.**
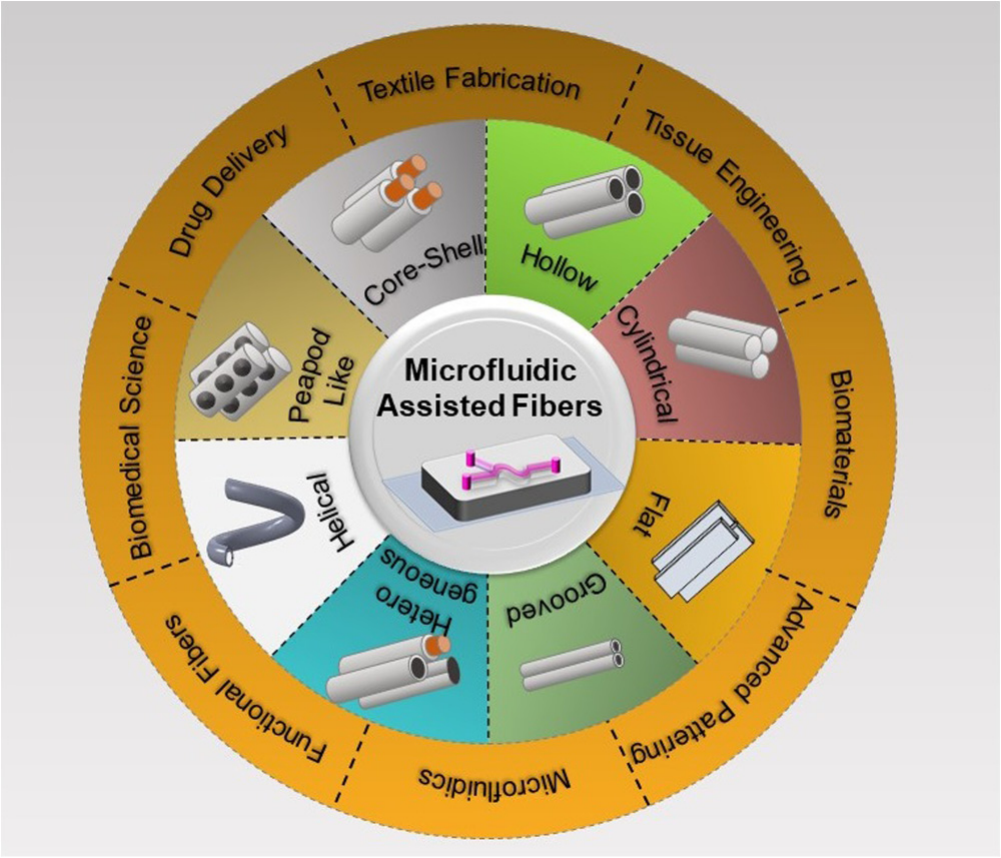
Overview of various shapes/morphologies of microfibers produced by microfluidic-assisted approaches (middle ring) and their potential applications (outer ring).

Microfluidic technologies have shown significant potential as novel platforms for controlled production of well-designed micro- and nano-structures with diverse shapes and geometries, such as particles,^30^ discs,^31^ capsules,^32^ fibers,^33^ and tubes.^34^ Of particular note, fibers produced using microfluidic techniques have been shown to be efficient for application in textiles,^35^ tissue engineering,^36^ drug discovery,^37^ biomedical science,^38^ advanced patterning,^39^ biomaterials,^40^ and functional fibers,^41^ among others. To date, researchers have made remarkable efforts to develop novel microfluidic approaches to produce fibrous materials with tunable shapes and diverse applications. However, a comprehensive review summarizing the recently explored microfluidic-assisted techniques for making fibrous structures aimed at different applications and their great promises and potential for future investigations is currently lacking in the literature. To bridge this gap, the present article seeks to provide a systematic review of microfluidic-assisted fiber production methods, elaborating on their state-of-the-art, developed technologies, targeted applications, and limitations and prospects (Fig. 1). In brief, first, the recent advances in the microfluidic approaches developed using different materials and techniques for fiber production are discussed, including capillary-based devices, microfluidic chips, three-dimensional (3D) printed devices, and devices made of tubes, pipets, and/or needles. Then, major materials and cross-linking mechanisms employed for the microfluidic-assisted fabrication of fiber-like structures are summarized in addition to the various shapes, morphologies, and characteristics the produced fibers exhibit. Additionally, various practical uses for the micro- and nano-fibers fabricated using microfluidic methods are highlighted in applications, such as biomedical sensors, tissue engineering, drug delivery, wearable electronics, and optical sensors. Finally, recent progress, current challenges, and future perspectives of the microfluidic-based technologies for the fabrication of fiber-like materials are outlined with their prospects for widening their application range and improving their performance.

## II. MICROFLUIDIC-BASED TECHNIQUES FOR THE PRODUCTION OF FIBERS

With excellent manipulation of microflows and without the need for complicated devices, microfluidic technologies have recently shown significant potential for producing micro- and nano-scale fiber structures for various applications. In this section, first, the principles of microfluidic systems are described briefly; then, the microfluidic strategies employed for fiber production are explained from the perspective of their platforms and geometries. Subsequently, the influence of the microchannels design and flow parameters on the characteristics of the produced fibers are discussed in detail. In particular, we highlight that the designed microfluidic platforms can produce a comprehensive collection of fibers with diverse structures, such as cylindrical, grooved, flat, anisotropic, hollow, core–shell, Janus, heterogeneous, helical, peapod-like, and knotted fibers with highly tunable sizes, and mechanical properties such as Young's moduli and porosities for variety of applications.^42^

### A. Principles of microfluidic systems

Microfluidic systems typically operate under laminar flow conditions (i.e., at low Reynolds numbers), providing superior control over the synthesis process. Therefore, the structure and functionality of the produced fibers can be finely tuned by manipulating the flows in microchannels, which are affected by surface tension and fluid viscosity, and are mixed only through diffusion at the interface between flows under laminar flow conditions.^43^ In a typical microfluidic fiber fabrication process, according to the arrangement of microchannels, sample fluid(s) consisting of the polymer precursor(s) and, if any, sheath solution(s) (i.e., non-polymerizable fluid) are introduced into separate input ports, and “core–sheath” flow profile is generated. In these approaches, the outer liquid has a vital role in carrying the inner liquid, shaping up the geometry of the inner fluid, preventing the inner fluid from coming into contact with the channel walls, and avoiding the clogging of the channel after fiber formation, among others.^44,45^ The size and shape of the final fibers could be precisely modulated by the flow rates of different fluids and the dimensions, shapes, and arrangements of the microchannels. Notably, the fluid solidification process must be rapid enough to enable the control and restoration of the desired fiber shapes.^46^ However, controlling the flow of fluids with high viscosity in a fluidic microchannel is challenging since the solidified fibers need to be easily extrudable without clogging the microchannel.^47^

Another critical point is that most of the fluids in these systems are non-Newtonian with a shear-rate-dependent viscosity, which must be considered while designing the microchannels for the use of these fluids. However, during the core–sheath flow formation and passage through the microchannel, the shear stress is minimal in most microfluidic fiber fabrication systems, which offers a suitable condition for preparing cell-laden microfibers.^8–48^

Therefore, a broad range of advantages of the microfluidic systems, such as energy-saving, cost-effectiveness, high surface to volume ratio, rapid mass/heat transfer, precise controllability, and the possibility of continuous production, enables these systems to produce micro/nanoscale fibers with diverse structures and functionalities.^43–49^ Furthermore, due to their flexibility, the spatiotemporal control over the safe loading of multiple components is also possible.^50^

### B. Microfluidic platforms

The microfluidic devices reported for fiber production have often been developed using either (cylindrical or rectangular) microchannels made of polydimethylsiloxane (PDMS) or thermoplastic polymers such as Cyclic Olefin Copolymer (COC) and poly(methyl methacrylate) (PMMA), pulled glass capillaries, three-dimensional (3D) printed devices, metal needles, or polymeric tubes (as summarized in Table I).^36–38^

**TABLE I.** Diverse microfluidic platforms/techniques as well as the materials and cross-linking agents used for the fabrication of fibers with various shapes/morphologies and sizes for a broad range of applications.

Microfluidic platform	Platform material	Platform/fabrication technique	Fiber material	Crosslinking agent/mechanism	Fiber shape/morphology	Fiber size (*μ*m)	Application(s)	Reference
Microfluidic chip	PDMS	Hydrodynamic focusing, core–sheath	Alginate	CaCl_2_ in PEG, ionic	Ribbon	5–25	Cell encapsulation, regenerative medicine, tissue engineering, therapeutic implantation	42
		Hydrodynamic focusing, core–sheath	PVA	Ethanol (EtOH), phase inversion	Ribbon	5–25	Cell encapsulation, biomedical applications	80
		Hydrodynamic focusing, core–sheath	PCL	PEG in EtOH–H_2_O mixture, phase inversion (solvent extraction)	Round, micro-fibrous scaffolds	2.6–36.5	Tissue engineering and regenerative medicine	121
		Hydrodynamic focusing, coaxial flow	PCL	PEG in EtOH–H_2_O mixture, phase inversion (solvent extraction)	Ribbon	13.3–33.65	Tissue engineering and drug delivery	84
		Hydrodynamic focusing, core–sheath	Gelatin	EtOH, coagulation bath	Round, square, and ribbon	30–282	Tissue engineering and drug delivery	122
		Hydrodynamic focusing, with grooves	Thiol-ene and thiol-yne	UV (photopolymerization), covalent bonding	Round and ribbon	50–110 & 125–330	Sensors, filtration, and textiles	92
		Hydrodynamic focusing, with grooves	4-hydroxybutyl acrylate (4HBA) and acrylic acid	UV (photopolymerization)	Flat and ribbon	12–198	Wound healing, controlled release materials, tissue engineering, and anti-ballistic textiles	94
		Y-shaped	Methacrylated hyaluronic acid (MA-HA) or chondroitin sulfate (MA-CS), alginate, and chitosan	CaCl_2_ (ionic), UV (photopolymerization), and coagulation bath	Round, multicomponent	546.06 ± 9.6 and 812.58 ± 79.57 and 1000	Tissue engineering (tendon)	106
		Hydrodynamic focusing, microfluidic spinning	Silk nanofibers	EtOH, coagulation bath	Round (aligned hierarchical)	∼20–55	Tissue engineering, regenerative medicine (nerve and blood vessel)	81
		Microfluidic spinning	Methacrylamide-modified gelatin	EtOH, coagulation bath, thermal solidification	Grooved	∼500	Tissue engineering, regenerative medicine	102
		Hydrodynamic focusing, microfluidic spinning	Alginate	CaCl_2_ (ionic)	Flat, grooved	30–100 x ∼1–30	Regenerative medicine, cell culture, and biomedical engineering	83
		Hydrodynamic focusing, microfluidic spinning	Alginate	Isopropyl alcohol (IPA) or IPA containing CaCl_2_ ⋅ 2H_2_O, ionic	Thin sheets, seaweed-like, semi-cylindrical, and cylindrical	0.07–20	Tissue engineering, biomedical engineering, and textiles	41
		Inertial microfluidics	Poly(ethylene glycol) diacrylate (PEGDA)	UV (photopolymerization)	Diamond, triangular, hollow, and U-shaped	∼120–320	Tissue engineering and textiles	7
		Y-shaped, core–shell	Graphene oxide (GO) and generation 3 polyamidoamine dendrimer-coated polystyrene	Annealing (hydrothermal process)	Round	220	Energy storage	123
		Hydrodynamic focusing	1,3,5-tris(4-aminophenyl)benzene and 1,3,5-benzenetricarbaldehyde	Schiff-base reaction (covalent bonding)	Round, sponge-like	0.07	Advanced patterning strategies such as 2D and 3D printing	10–39
		Coaxial flows	Alginate	CaCl_2_ (ionic)	Hollow	410–500	Endothelial barrier research and drug testing	37
		Flow focusing, droplet-based microfluidics	Alginate	CaCl_2_ (ionic)	N/A	∼15–50 *μ*m	Tissue engineering, biofabrication, biomedical engineering	86
		3D device, solution blow spinning	Perfluorocopolymer or PCL	Solvent evaporation	Round	1.4–4.2	Bio-based nanofibrils, textiles, air filtration, drug delivery, wound dressings, and tissue engineering	12
		Hydrodynamic focusing, microfluidic spinning	Collagen type I	PEG, pH-induced solidification	Round	3–10	Biomedical engineering, tissue engineering, and suture- or wound-dressing materials	45
		3D hydrodynamic flow focusing, microfluidic wet spinning	PU, poly(acrylonitrile) (PAN), and PVA	PEG, coagulation bath	Round, ribbon	4–154	Textiles, nonwoven fabrics, and injection molding	103
		Coaxial flows, microfluidic spinning	Poly(l-lactic-co-ε-caprolactone) (PLCL)	Methanol, precipitation of PLCL, coagulation bath of methanol	Round and irregular	12–36	Biomaterials, surgical sutures (ophthalmology suture)	40
		Valve-based device, coaxial flows, microfluidic spinning	Alginate	CaCl_2_ (ionic)	Spindle-knots and gas micro-bubbles	20–220	Biosensors, high-throughput screening, and tissue engineering	124
		Coaxial flows, core–shell	Alginate	CaCl_2_ (ionic)	Round	∼100	Functional fibers and tissue engineering	91
		Hydrodynamic focusing	Alginate	CaCl_2_ (ionic cross-linking) + UV (photopolymerization)	Solid, hollow, hollow double-layered, osteon-like	∼450	Tissue engineering and biomedical research	48
		Cylindrical and coaxial-flow channels	Collagen-alginate	CaCl_2_ (ionic)	Round	250	Enhanced immunoprotection of transplanted islets, tissue engineering	82
		Core–sheath, microfluidic spinning	Regenerated silk fibroin (RSF) and CaCl_2_	Dry spinning, solvent evaporation	Round	5–10	High-performance artificial animal silks and synthetic fibers	85–125
		Core–sheath, microfluidic wet-spinning	Recombinant spider dragline silk protein	EtOH coagulation bath, solvent evaporation	Round, ribbon	1–5	Artificial fiber with high strength	126
		Core–sheath, microfluidic spinning	RSF and silk sericin (SS)	Dry spinning, solvent evaporation	Core–shell, grooved, and spindle-knot	∼10–20	Humidity sensors, drug delivery, and lustrous silk-like fabric	87
		Co-flowing, core–shell	PEGDA	UV (photopolymerization)	Single- and double-hollow, microbelts, and occluded-, acorn-, and heteroaggregate-shaped	57–77	Complex-shaped composites, encapsulation of active agents, cell culture, catalysts support, affinity membranes, hierarchical filter materials, and protective clothing	79
		Three-dimensional flows, multi-layer device, core–shell	Alginate	CaCl_2_ (ionic), coagulation bath	Multi-compartmented and multi-layered circular, non-circular, asymmetric core–shell, and hollow fibers	∼100–150	Functional fiber materials, medical applications, biological research, co-culture of several types of cells	108
		Hydrodynamic focusing, microfluidic spinning	Alginate and propylene glycol alginate (PGA)	CaCl_2_ (ionic)	Anisotropic rounded rectangle	7–200	Tissue engineering	88
		Microfluidic solution blow spinning	Dyneon THV 221 GZ	Solvent evaporation	Round	0.036–0.062	Drug delivery and tissue engineering	89
		Flow focusing, droplet-based microfluidics	PEGDA	UV (photopolymerization)	N/A	∼<15 *μ*m	Tissue engineering, cell-based studies	127
		Flow focusing, microfluidic spinning	Alginate and gelatin methacrylate (GelMA)	CaCl_2_ (ionic), UV (photopolymerization)	Grooved	290.29 ± 10.16–437.22 ± 7.36	Tissue engineering, regenerative medicine	128
		Hydrodynamic focusing, microfluidic wet spinning	RSF and cellulose nanofibers (CNFs)	Coagulation bath, solvent exchange	Round, ribbon-like	∼23–35	Biophotonics, photothermal therapy, medicine delivery, visible biological scaffolds	129
		Hydrodynamic focusing, microfluidic dry spinning	RSF and CNF	Dry spinning, solvent evaporation	Round	7.9 ± 2.0–8.4 ± 1.1	High-performance artificial fibers	130
		Flow focusing, microfluidic wet spinning	RSF and CNF	Coagulation bath, wet spinning	Deformed round	∼20–50	Highly oriented artificial fibers, tissue engineering	131
		Hydrodynamic focusing, core–sheath, microfluidic spinning	Alginate	CaCl_2_ (ionic)	Multicomponent heterogeneous	∼200–400	Cell culture, tissue engineering, composite functional biomaterials, biomimetic systems	132
		Flow focusing, microfluidic wet spinning	Alginate, PAN, and regenerated B. mori silk	CaCl_2_ (ionic), H2O-DMSO (non-solvent-induced phase separation), and EtOH in PEG (coagulation)	Round, grooved	0.8–10	Tissue engineering	133
	PMMA	Hydrodynamic focusing, microfluidic spinning	GO and alginate	CaCl_2_ (ionic), Coagulation bath of EtOH-H_2_O, hydrodynamically induced macromolecules alignment	Skin-core, eccentric structure, sandwich shapes, and reverse core shaped	80–100	Wearable devices and actuators, smart textiles	96
		Hydrodynamic focusing	PEGDA	UV (photopolymerization)	Hollow	120–124	Synthetic human vasculature, tissue engineering	134
		Hydrodynamic focusing, microfluidic spinning	Alginate	CaCl_2_ (ionic), coagulation bath	Ribbon, grooved	29.7–69.7	High performance and multi-layered fibers	97
		Flow focusing, multi-layer device	Alginate	CaCl_2_ (ionic)	Round	177.4–387.5	Drug release (delivery) and cell encapsulation	99
		Hydrodynamic focusing	Alginate, PEG, and gelatin	CaCl_2_ (ionic), coagulation bath	Hollow	∼400–650	Cell encapsulation and tissue engineering	135
		Hydrodynamic focusing	Mixture of liquid crystal mesogens (MAOC4 and MACC5)	UV (photopolymerization)	Ribbon	10 ± 3–172 ± 8	Biomaterials, multi-functional materials, and optical fabrics	101
	Glass	Hydrodynamic 2D and 3D flow focusing, microfluidic wet spinning	Gellan Gum and GelMA	CaCl_2_ (ionic), coagulation bath	Round (core–shell), ribbon-like, double-Janus, tri-coaxial, double Core-Shell, peapod-like	∼50–400	Tissue engineering, regenerative medicine	136
	PDMS-Glass	Y-shaped, microscope-based photolithography	PEGDA	UV (photopolymerization)	Square or rectangular	5–30	Biomedical applications, drug delivery, and micro-filtration devices	137
	PDMS-plastic capillary tube	Y-shaped, coaxial flows	Alginate	CaCl_2_ (ionic)	Hollow	600–650	Immobilization of enzymes	107
	PDMS-polyether ether ketone (PEEK)	Hydrodynamic focusing, with grooves	Thiol-ene prepolymers	UV (photopolymerization)	Double anchor-shaped	780 × 300	Textiles, composite reinforcement, tissue engineering, filtration	138
	PDMS-nylon mesh filter	Coaxial flow, core–sheath, microfluidic spinning	Collagen and alginate	CaCl_2_, ionic	Round, multicomponent	∼100	Tissue engineering and cell encapsulation	139
	Aluminum-stainless steel-poly(methyl methacrylate) or Kapton	Hydrodynamic focusing, microfluidic wet spinning	CNF and cellulose nanocrystals (CNCs)	pH-induced solidification	Ribbon and elliptical	∼10–25	Bio-based advanced materials, industrial production of fibers	11
	Aluminum-stainless steel-poly(methyl methacrylate) (PMMA) or Kapton	Hydrodynamic focusing, microfluidic wet spinning	CNF	pH-induced solidification	Round	∼20	Templates or high-performance filaments	140
	Aluminum-poly(methyl methacrylate) (PMMA)	Hydrodynamic focusing, microfluidic spinning	CNF	Coagulation bath, electrochemical	Round, hollow	∼20–40	Bio-based materials, high-performance bio-composites, and textiles	141
	Teflon-aluminum	Hydrodynamic focusing, with grooves	PMMA	Fructose solution, solvent exchange	Flat and ribbon	0.3 to >5	Anti-ballistic textiles	93
	Aluminum-PDMS-cyclic olefin copolymer (COC)	Hydrodynamic focusing, with grooves	Thiol-ene and thiol-yne	UV (photopolymerization)	Round, flat, square, and complex	0.3–1000	Tissue engineering, optical communications, and smart textiles	8
Capillary-based device	Glass	Coaxial flow, core–shell	Alginate and PVA	CaCl_2_ (ionic), non-solvent-induced phase separation (NIPS) process	Hourglass-shaped, round	∼50–300	Dehumidification and water collection	54
		Coaxial flow, core–shell	Alginate	CaCl_2_ (ionic)	Peapod-like, cylindrical rods, conical frustums, barrels, and plates	20	Drug delivery, biomedical therapeutics, microlenses, and encapsulation of biomaterials	53
		Microfluidic spinning, core–shell	Bovine serum albumin (BSA) and Glutaraldehyde (GA)	Schiff-base reaction (covalent bonding)	Round	23–30	High-performance biological fibers	142
		Vertical coaxial flows, core–sheath	Paraffin Rubitherm®27 (RT27) and Poly(vinyl butyral) (PVB)	Solvent extraction, phase change	Hollow fiber	330–400	Textile, aviation, military, and healthcare	55
		Coaxial flows, core–sheath	RT27 and PVB	Solvent extraction, phase change	Hollow	∼300–400	Textiles, aviation, military, and space	60
		Coaxial flows, microfluidic spinning	Alginate, GO, and polyacrylamide	CaCl_2_ (ionic)	Round	200–600	Tissue engineering (muscles), actuators, and sensors	64
		T-junction, microfluidic spinning	Alginate and PLA	CaCl_2_ (ionic), coagulation bath	Peapod-like (bead-on-string fibers)	∼180–600	Wound healing, biomedicine, and tissue engineering	143
		T-junction, microfluidic spinning	Thermoplastic PU, black phosphorous, and carbon nanotubes	Solvent exchange	Round	80	Wearable electronics, energy storage	144
		T-junction, microfluidic spinning	PCL and formic acid	H_2_O (coagulation), physicochemical phase inversion	Helical	∼100	Tissue engineering and wound healing	145
		Coaxial flows, microfluidic wet spinning, core–sheath	Cis-1,4-polybutadiene or cis-1,4-polyisoprene	Coagulation bath, solvent extraction	Round	25–200	Smart textiles, wearable sensors, and robotic actuators	51
		Coaxial flows	Alginate-collagen	CaCl_2_ (ionic)	Mono-, double- and triple-layer helical, hollow, superhelical	∼100–400	Blood vessel-on-a-chip, biomimetic applications	72
		Coaxial flows, consecutive spinning, and spiraling	Alginate	CaCl_2_ (ionic)	Hollow, core–shell, and double-helical	∼<100	Intelligent microsprings, biosensors	59
		Coaxial flows, square and cylindrical capillary tubes	Polyethersulfone (PES)	PEG, phase inversion	Hollow	∼600–850	Hollow fiber membranes	73
		Coaxial three-phase flow device	Chitosan and acetic acid	GA (covalent bonding)	Tubular and peapod-like	∼120–580	Tissue engineering, cell culture, wound healing	74
		Coaxial flows, core–sheath, cylindrical capillaries, and square tubes	Alginate and sodium carboxymethyl cellulose (CMC)	CaCl_2_ (ionic), coagulation bath	Hollow	45.6–175.5	Cell encapsulation, cell culture, and functional microfibers	62
		3D coaxial flows, microfluidic spinning	Hydrophilic carboxylated chitosan (CCS) or PVA or PU-urea (PUU3-12)	PEG, solvent diffusion (exchange)	Helical	∼25–75	Smart devices and flexible sensors or actuators	76
		Core–shell, microfluidic spinning	PU and liquid metal (LM)	EtOH bath, coagulation	Hollow	100–500	Wearable electronics	77
		Coaxial flows, core–shell, droplet-based microfluidics	Alginate and GelMA	CaCl_2_ (ionic), UV (photopolymerization)	Spindle-Knotted	∼50–500	Humidity-responsive water capture, thermally triggered water convergence, colloidal crystal assembly, and cell microcarrier arrays	57
		Co-flowing, coaxial flows, hydrodynamic focusing	Alginate and poly (3,4-ethylenedioxythiophene) (PEDOT), poly (4-styrenesulfonate) (PSS)	CaCl_2_ (ionic), acid treatment, coagulation bath	Round, core-shell	∼200–300	Flexible electronics, sensors, and wearable systems	58
		Coaxial flows	Acrylamide, alginate, PEDOT:PSS	*N,N*-methylenebisacrylamide (MBAA) (covalent), CaCl_2_ (ionic), UV (photopolymerization)	Round	715 *μ*m	Strain sensors, wearable and implantable sensors, soft robotics	146
	Glass-PTFE	3D coaxial flows	Glycidylmethacrylate-dextran (GMA-dex)	PEG (solvent diffusion (exchange)), UV (photopolymerization)	Helical	18–50	Biomedical engineering, adaptive microdevices	147
	PMMA-Glass	Coaxial flows, flow focusing	Gelatin-alginate	CaCl_2_ (ionic), coagulation bath	Straight and helical hollow	210–310 and 400–600	Biomimetic scaffolds, controlled release, cell encapsulation	70
	Silica-Glass	T-junction, co-flowing	4′′-acryloyloxybutyl) 2,5-di(4′-butyloxybenzoyloxy) Benzoate and benzyldithiobenzoate	UV (photopolymerization)	Round	3–75	Actuators, biosensors, microelectromechanical systems, and active surfaces	71
	Glass-stainless steel-epoxy resin	Coaxial flows, core–sheath	Chondroitin sulfate C and chitosan	Polyion complex formation (molecular self-assembly in aqueous media)	Round (solid) and hollow	236 ± 18 (solid) 168 ± 20 (hollow)	Tissue engineering, biomedical engineering	65
	Glass-pinhead	Double coaxial flows	Alginate - GelMA	CaCl_2_ (ionic) + UV (photopolymerization)	Double-layer hollow	195–535	Tissue engineering	61
	Polycarbonate	Coaxial flow	Alginate	CaCl_2_ (ionic)	Round	300–550	Food engineering	112
	Polytetrafluoroethylene tubes	Coaxial flows, core–sheath, microfluidic spinning	Alginate and thermochromic powder	CaCl_2_ (ionic), coagulation bath	Round	∼200–300	Smart clothing, textile	148
3D printed device	3D printing resins, catheter, and Optran UV fiber	Y-shaped, with tubes and optical fiber, microfluidic spinning	Alginate with tantalum nanopowder (DAT)	CaCl_2_ (ionic cross-linking) and visible light irradiation (covalent)	Round	213 ± 24–635 ± 30	Aneurysm embolization	149
	Photoreactive resin	Co-flowing, modular	Collagen-alginate solution	CaCl_2_ (and sucrose), ionic	Round	100–300	Analytical chemistry, biology, and tissue engineering	109
	Photoreactive materials and polycarbonate	Hydrodynamic focusing, extrusion-based deposition	Carbon microfiber (CMF), N-isopropylacrylamide	MBAA (covalent), UV (photopolymerization)	Round	1830 ± 100	4D printing	150
	Glass-photoreactive resin	With embedded capillaries, coaxial flows	Alginate	CaCl_2_, ionic	Helical	100–400	Micro-sensors, soft actuators, and medical applications	50
Pipet- and needle-based device	Blunt needle-stainless needles-pipet tip-glass	Coaxial flows, microfluidic spinning	Alginate	CaCO_3_ + CaCl_2_ (ionic), coagulation bath	Hollow knotted microfibers	200–950	Bioengineering, biomedical applications, and drug testing	151
	Blunt needle-stainless needles-pipet tip-glass	Coaxial flows, microfluidic spinning	Alginate	CaCl_2_ (ionic), coagulation bath	Spindle-, hemisphere- and petal-knotted, hollow	∼50–1000	Bioengineering, biomedical applications, and tissue engineering	152
Needle-based device	Metal needle-plastic tube	Coaxial flow, microfluidic wet extrusion	Collagen type I	EtOH bath, pH-induced solidification	Ribbon	31.1 ± 1–46.4 ± 2 × 9.2 ± 0.5–11.9 ± 0.5	Surgical suture and biotextile manufacturing (biotextiles)	153
Microfluidic tubular device	PTFE and stainless steel	T-junction, droplet-based microfluidics, microfluidic spinning	Polychromatic PLA, alginate, PVA	PEG, coagulation bath	Beaded fibers, hourglass-shaped, ribbon	100–700	Anti-counterfeiting, military camouflage, and wearable displays	35
Pulled pipets with PDMS molding	PDMS-glass	Coaxial flows, core–sheath	4HBA, PVA, and acrylic acid	UV (photopolymerization)	Round fibers and microtubes	20–90	Biosensors, actuators, tissue engineering, and drug delivery	154
		Embedded capillary glass pipet, core–sheath	Alginate	CaCl_2_ (ionic), coagulation bath	Round	19–55	Tissue engineering, regenerative medicine (nerve or muscle), actuators, and sensors	47
		Embedded capillary glass pipet, coaxial flows	Poly(ethylene oxide)	EtOH, Solvent extraction	Round	2–6	Electronics, tissue engineering, and drug delivery	44
		Core–sheath flows	Chitosan in acetic acid	Sodium triphosphate pentabasic (STP), ionic	Round	70–150	Liver tissue formation, tissue engineering	66
		Coaxial flows, microfluidic wet spinning	Chitosan-alginate	CaCl_2_ (ionic)	Round	65–135	Tissue engineering, cell encapsulation, and analyzing cellular behavior in three-dimensional (3D) structure	67
Double-syringe injection device	Luer-Lok fittings and polypropylene tubing	Hydrodynamic focusing	Carboxymethyl chitosan (CMCh)	Zinc nitrate hexahydrate (Zn(NO_3_)^2^ ⋅ 6H_2_O) (ionic)	Round and elliptical	731.4–888.8	Biomedical engineering and wound dressing material	110

**FIG. 2.**
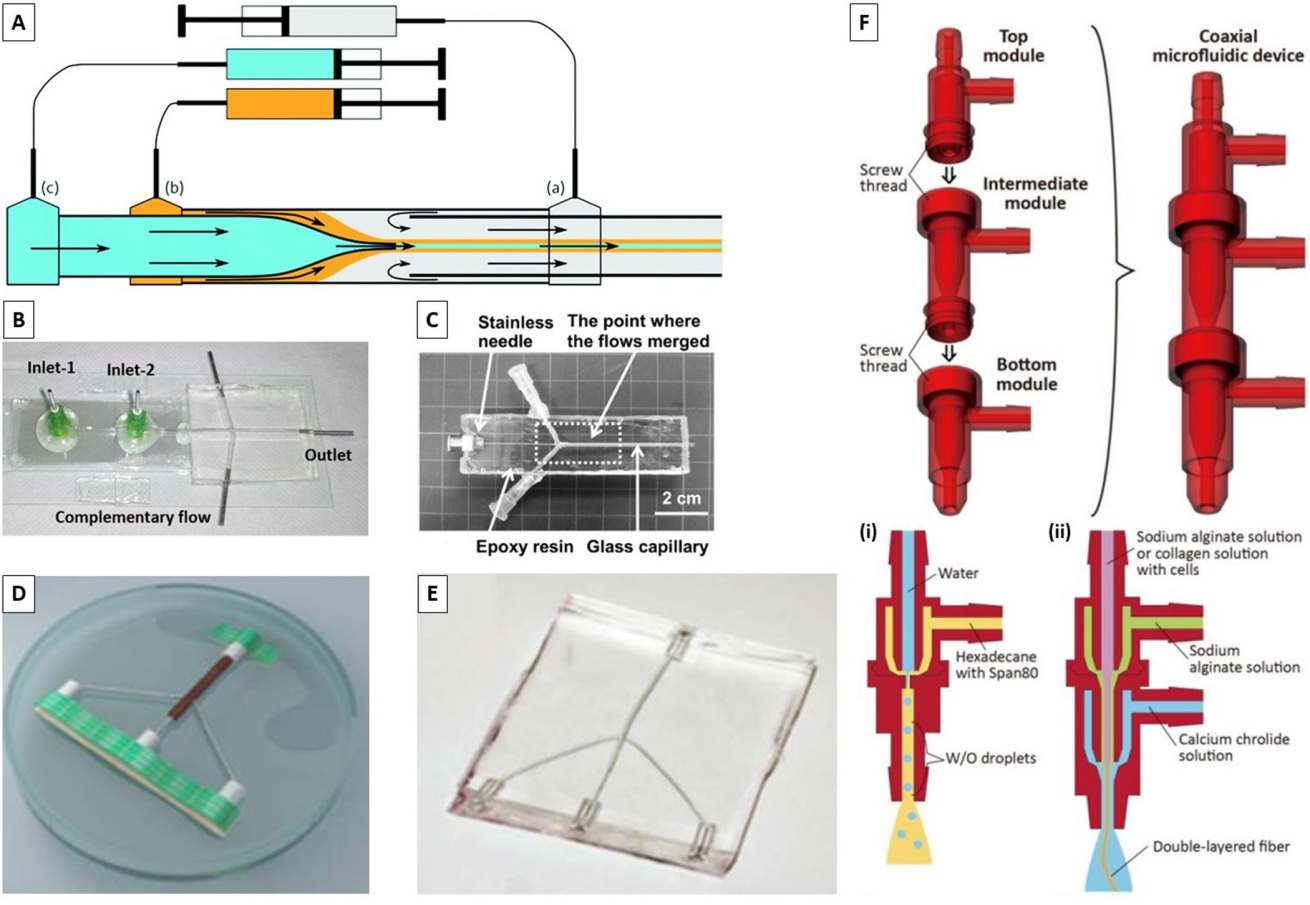
Different microfluidic platforms. (a) Pulled glass capillaries. Reproduced with permission from Honaker *et al.*, J. Mater. Chem. C **7**(37), 11588–11596 (2019).Copyright 2019 Royal Society of Chemistry. (b) Glass capillaries in PDMS. Reproduced with permission from Pullagura and V. Gundabala, Langmuir **36**(5), 1227–1234 (2020). Copyright 2020 American Chemical Society. (c) Stainless needle and glass capillaries in PDMS. Reproduced with permission from Iijima *et al*., Polym. J. **50**(12), 1187–1198 (2018). Copyright 2018 Elsevier. [(d) and (e)] Embedded template method in PDMS. Reproduced with permission from Pham *et al.*, J. Appl. Phys. **117**(21), 214703 (2015). Copyright 2015 American Institute of Physics. (f) 3D printed modules for the coaxial microfluidic device and its application to produce (i) W/O droplets using water and hexadecane and (ii) a double-layered alginate gel fiber. Reproduced with permission from Morimoto *et al.*, Sens. Actuators, B **274**, 491–500 (2018). Copyright 2018 Elsevier.

The glass has several advantages such as surface hydrophilicity, circular cross section to form stable coaxial flow, surface modification by hydrophilic or hydrophobic coating to work a variety of materials, and pulling using a pipet puller and cutting to different orifice diameters [Fig. 2(a)].^36–51^ Because of these advantages, many researchers have made their microfluidic devices with diverse structures from simple core–shell^52,53^ to multiple-core^54–59^ and multilayered structures^60–63^ by pulled or regular glass capillaries using PDMS [Figs. 2(b) and 2(c)],^44–69^ plastic,^70^ or glass^50–78^ support channel. The glass capillary-based systems are relatively low cost, simple, and rapid; however, they are labor-intensive, have poor reproducibility, are based on time-consuming procedures, and require skilled personnel and specialized tools (i.e., microforge or microcapillary puller).^36–79^

Alternatively, two-dimensional (2D) microfluidic chips can be fabricated through soft lithography using elastomeric materials (such as PDMS)^10–91^ and computer numerical control (CNC) or milling machine using thermoplastic materials (e.g., PMMA), fluoropolymers [e.g., polytetrafluoroethylene (PTFE)], or metals.^8–101^ These systems offer several advantages, including high reproducibility, bio/chemical compatibility, low production costs, precisely designed microchannels, and fast prototyping. Most microfluidic devices fabricated following the conventional soft lithography technique have microchannels with a rectangular cross section. However, several researchers have proposed novel methods to make channels with cross sections other than rectangular. Shi *et al.* proposed a method to generate PDMS devices with well-defined longitudinal grooved cylindrical channels to generate fibers to efficiently direct the alignment of cells grown on them, favoring anisotropic tissue formation.^102^ Jun *et al.* successfully fabricated cylindrical channels with different dimensions and used oxygen plasma bonding to make the coaxial flow via two aligned hemi-coaxial flow channels.^82^ Nguyen *et al.* fabricated a microfluidic flow-focusing device with cylindrical microchannels using a combination of micromachining and replication molding without employing a complex glass microcapillary.^37^ In this method, two PDMS replicas with a semi-cylindrical microchannel were aligned to obtain a circular microchannel with mild and continuous coaxial flows to fabricate hollow fibers.^37^

On the other hand, producing microfluidic devices via replica molding against a master mold fabricated by soft lithography is relatively inexpensive; however, this technique requires an initial investment in necessary cleanroom facilities. Although the resolutions and tolerances of the microfluidic devices made by additive manufacturing techniques are lower than those made by soft lithography, these methods were recently investigated for rapid and cost-efficient prototyping.^103–105^ Costa-Almeida *et al.*^106^ and Gursoy *et al.*^103^ presented a templating method based on PDMS elastomers combined with an array of disposable stainless-steel hypodermic needles that may be removed after the PDMS curing. Their devices can achieve precise three-dimensional (3D) hydrodynamic focusing with laminar flow conditions.^103–106^ Pham *et al.* demonstrated a straightforward and cost-effective method based on the embedded template to fabricate PDMS microfluidic devices without requiring specialized facilities. As such, a diverged Y-shape template platform with three inlet ports and one outlet was fully covered by the PDMS pre-polymer. After curing the PDMS, the templates were pulled out and the voids were created in the device, giving rise to the microfluidic channels [Figs. 2(d) and 2(e)].^107^

Recently, several 3D microfluidic spinning chips consisting of multiple PDMS layers containing microchannels have been fabricated for the formation of highly heterogeneous microfibers.^108^ However, the production time for larger and complicated coaxial channels is prolonged, the yield is reduced, and rinsing and reusing the inside of the channels is difficult. To tackle these problems, modular microfluidic approaches have been proposed [Fig. 2(f)].^109^ In this vein, Morimoto *et al.* developed a coaxial microfluidic device by connecting the 3D printed microfluidic modules via screw threads.^109^ However, most of the reported modular microfluidic devices have complicated fabrication processes and, thus, are not suitable for large-scale production. To resolve this drawback, Wang *et al.* managed to reduce both the complexity and the cost of a designed double-syringe injection device built using a syringe pump and some commercial Luer-Lok fittings and polypropylene tubing.^110^

### C. Microfluidic strategies for fiber production

#### 1. Core–sheath flow geometries

**FIG. 3.**
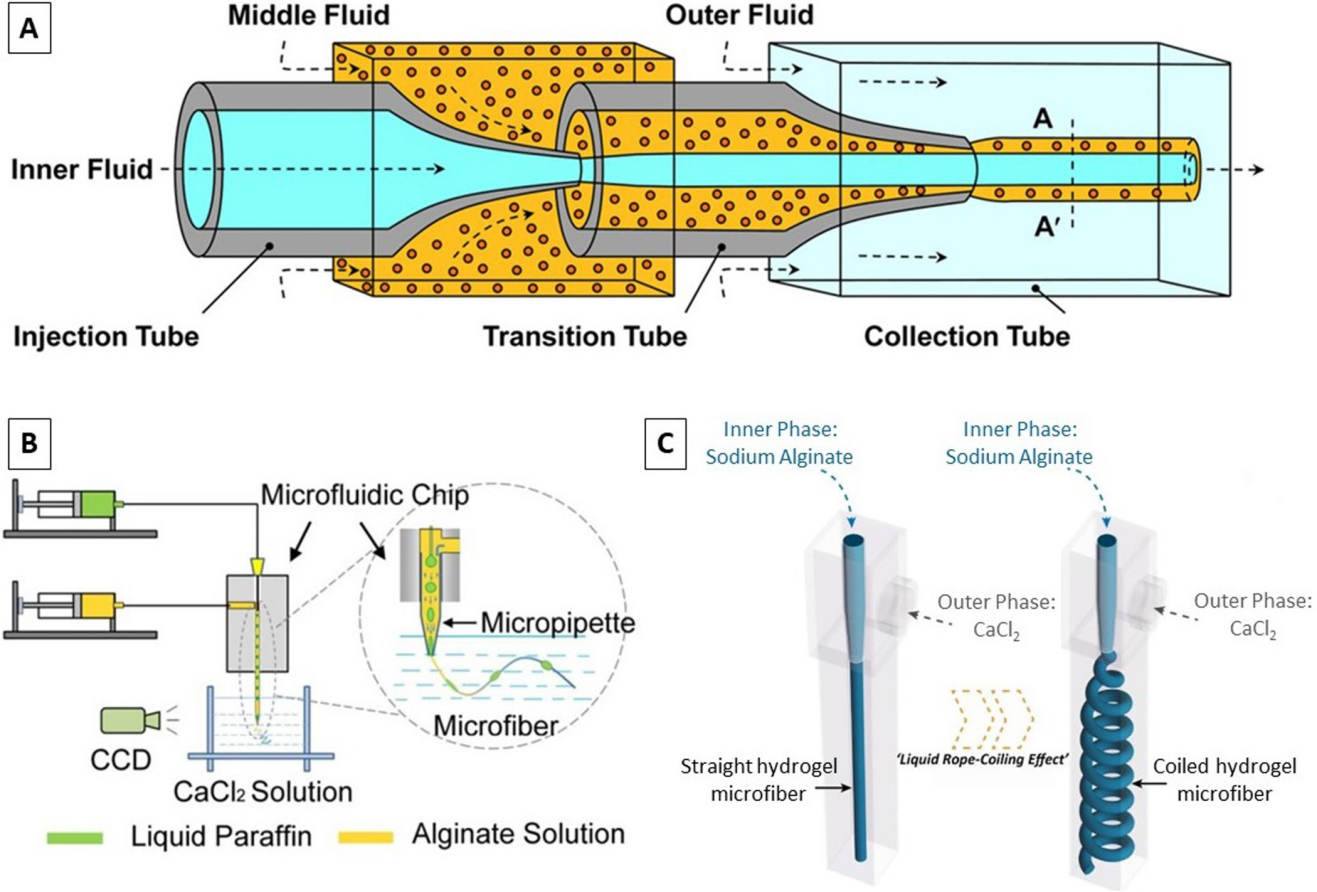
(a) Typical core–sheath (co-flow) flow geometry. Reproduced with permission from Zhang *et al.*, J. Mater. Sci. **53**(23), 15769–15783 (2018). Copyright 2018 Springer Science Business Media, LLC, part of Springer Nature. (b) Schematic diagram of the experimental process for the preparation of fibers with spindle-knots. Reproduced with permission from Ji *et al*., RSC Adv. **5**(4), 2517–2522 (2015). Copyright 2015 Royal Society of Chemistry. (c) Schematic illustration of continuous microfluidic spinning and the “Liquid Rope Coiling Effect” in a microfluidic device. Reproduced with permission from Liu *et al*., Sens. Actuators, B **304**, 127069 (2020). Copyright 2020 Elsevier.

In 2004, Jeong *et al.* showed the first co-flow microfluidic device by glass capillary in PDMS microchannel to pass the core and sheath fluids.^111^ Their device has a cylindrical geometry, and by flowing the sample pre-polymer fluid in the core and not-polymerizable fluid in the sheath, the setup prevents the fiber-forming fluid from touching the device wall. They also reported the formation of hollow fibers by adding a secondary inert inner core flow within the polymerizable solution in the outer core and the sheath solution.^46^ In such devices with laminar regimes, i.e., fluid flows with low Reynolds numbers (Re), the radius of the central flow (Rs) can be determined based on the flow rates using the following equation:

$$R_{s}=R{\left[1-{\left[\frac{Q_{sheath}}{Q_{sheath}+Q_{sample}}\right]}^{\frac{1}{2}}\right]}^{\frac{1}{2}},$$

where *R* is the core channel radius, *Q*_sheath_ is the volume flow rate of the sheath flow, and *Q*_sample_ is the volume flow rate of the sample flow.^49^ According to this equation, the solid fiber diameter can be changed by varying the ratio of the input flow rates, without changing the device setup. The inner diameter and wall thickness of the hollow microfibers were altered by altering the core and sheath flow rates in the microfluidic channels.^107^ The experimental results showed that the outer diameter of the hollow fibers was independent of the flow rates, while their internal diameter and wall thickness were found to be a function of the core and sheath flow rates. At a fixed sheath flow, rising the core flow rate resulted in an increase in the internal diameter, while the wall thickness decreased. On the other hand, at a constant core flow, increasing the sheath flow led to a decrease in the internal diameter, with the wall thickness being increased.^107^ There are a large number of similar researches that produce cylindrical solid fiber,^11–112^ cylindrical hollow fiber [Fig. 3(a)],^37–113^ or cylindrical multilayer fibers^51–109^ with similar devices. Increasing the number of core flow channels and sheath flow channels, it is possible to get solid, hollow, and multilayer fibers with one device by adjusting proper fluids into different channels.^48–99^ There have been attempts to make non-cylindrical fibers with core–sheath flow geometry. Kang *et al.* engraved multiple groove patterns within the sample channel and fabricated thin flat fibers with longitudinal grooved patterns on the surface.^83^ The shape and size of the flat fibers and grooved patterns are affected by the flow rates of the sheath and sample streams. They showed that the thinner flat fibers could be achieved by slower sample flow rates or faster sheath flow rates; in addition, they proved that the sheath flow rate is more critical to reducing the thickness of the flat fiber than the sample flow rate.^83^

Recently, researchers developed various core–sheath flow microfluidic strategies for preparing microfibers with encapsulated gas bubbles,^53^ liquid droplets,^35–74^ or controllable multicompartmental internals (i.e., fibers with spindle-knot internals) [Fig. 3(b)].^54–100^ Shi *et al.* presented a novel microfluidic-based technique, through encapsulation of oil droplets in fibers, to generate bioinspired microfibers with hourglass-shaped knots via integrating a non-solvent-induced phase separation (NIPS) process.^54^ First, spindle-microfibers were partially gelled in ethanol, then the encapsulated oil cores leaked from the knots, and finally, the fibers with hourglass-shaped knots were produced. Moreover, Ji *et al.* have developed a simple coaxial microfluidic device with a micropipet installed at its outlet to fabricate alginate fibers with spindle-knots.^100^ The spindle-knots were formed due to the deformation and retraction process of the microdroplets at the constriction part of the micropipet. The two-phase flow rate ratio and the micropipet diameter were identified to be the key parameters for regulating the height, width, and interval of the spindle-knots as well as the diameter of the fibers.

To produce fibers with non-circular cross section, Nunes *et al.* developed an inertial microfluidics method with a user-friendly software design component.^7^ In this inertial process, “Re” is an important parameter and must be sufficiently large (i.e., Re > 8) to enable fiber production. There is a sequence of pillars in the device channel that sculpt the streams. The flow rate ratio of the three streams, i.e., two miscible outer non-reactive streams and one central reactive stream, determines the rectangular cross-sectional areas.

The formation of helical fibers in microfluidic channels is based on the “liquid rope-coil effect” phenomenon. In this phenomenon, when the viscous liquid strikes a surface under gravity, it initially shows a buckling behavior but eventually forms into stable coil-like patterns.^78^ Liu *et al.* could continuously and controllably fabricate helical hydrogel microfibers with flexible shapes by simply adjusting the flow rates in a coaxial microfluidic device [Fig. 3(c)].^50^ Their results showed that the diameters and shapes of the fibers could be manipulated by controlling the flow rates of phases under various viscosity contrasts of inner and outer fluids.^50^ The device, composition, or flow rates of internal and external fluid phases are vital parameters for fabricating various complex helical structures such as multilayer helical microfibers; straight hollow microfibers with straight, wavy, or helical channels; and super-helical hollow microfibers with straight or helical channels.^59–76^ Gao *et al.* produced cylindrical hollow calcium alginate microfibers with either straight or helical inner walls by a double coaxial flow microdevice.^70^ Their results demonstrated that the ratio of the outer to the inner diameter of a straight hollow fiber was inversely correlated with the combination of the flow rates of the core and first sheath streams. The helical pitch and spiral radius of the helical hollow microfibers were realized to be strongly influenced by the flow rate of the second sheath stream.^70^ On a similar topic, Pullagura and Gundabala employed a complementary flow toward the downstream end of the fiber solidification region to control both the fiber size and the extent of coiling of the generated fiber.^44^ Therefore, both non-woven and single fibers could be fabricated using the same device and variation of the complementary flow.

#### 2. No-sheath flow geometries

Although a majority of the research efforts have focused on advancing the microfluidic technologies having sheath flow streams, some researchers have also demonstrated the development of microfluidic systems without having any sheath flow steams for the fabrication of fibers.^102–114^ Among those, Costa-Almeida *et al.* produced multi-component hydrogel fibers with aligned structures using a combination of two widely used fiber fabrication methods, i.e., microfluidics and polyelectrolyte complexation. In this device, two oppositely charged polyelectrolyte solutions are injected into a Y-shaped microchip, enabling the formation of hydrogel fibers due to the electrostatic interactions between the solutions.^106^

Also, Li *et al.*^81^ and Peng *et al.*^85^ developed a bioinspired microfluidic chip with a geometry mimicking the natural silk gland. In this type of microfluidic spinning chip, the assembly and orientation of the protein molecules and fibrils are induced by the integrated shearing and elongational sections. The fibers generated using these devices presented an aligned hierarchical structure with the fiber mechanical properties superior to fibers derived from the traditional spinning approaches.^81^ Although the no-sheath flow geometries may result in increased ease in the experimentation owing to a smaller number of flows and required utilities, their microchannels are typically more likely to be clogged due to the lack of sheath flows to control the reaction rate inside the microchannel and, hence, they are more tricky to operate.

#### 3. Complex geometries

After introducing the core and sheath flows into the device [e.g., in a capillary-based device such as the one depicted in Fig. 2(a)], hydrodynamic focusing laterally focuses the core fluid into a thin vertical stripe that spans the height of the channel. The width of this stripe and the final cross-sectional area of the fiber after cross-linking are determined by the flow rate ratio between the sheath and core fluids.^92^ To make this device a 3D flow-focusing system, a series of recessed grooves are patterned into the floor and ceiling of the channel downstream of the initial focusing region.^42–94^ These grooves generate advection perpendicular to the channel axis such that the sheath fluid wraps around the core, focusing the core vertically and isolating the core fluid from the channel. Such a device can shape the fibers using different numbers of inlets and channel grooves (i.e., chevron, diagonal, and herringbones) with various designs.^8–101^ In addition, complex fluid shapes can be formed (e.g., round or ribbon-shaped cross sections) by varying the viscosity and hydrophilicity of the sheath and core fluids and minimizing the interfacial effects.^84–95^

Martino *et al.* designed a pulsatile microfluidic device to sequentially perform the segmentation of the alginate solution jet, which travels into the polymerization channel and meets a buffer stream containing Ca^2+^ ions to fabricate alginate fibers with lengths between 200 and 1000 *μ*m. Subsequently, the segmented fibers were encapsulated within pL-volume microdroplets using the same device. The advantage of this device is that the cutting can be done using pressures of only a few millibars without affecting the downstream flows.^86^

Following the introduction of solution blow spinning (SBS) in 2009,^115^ microfluidic scientists have also made use of this technique for fiber production. In this vein, recent progress in microfluidic technologies has paved the way for the fabrication of microfluidic devices to produce liquid microjets based on the gasdynamic virtual nozzle principle with unique control over the jet diameter and velocity. These microfluidic devices have been shown to enable the continuous fabrication of microfibers with excellent control over the fiber diameter and the internal crystalline alignment with the tuned mechanical properties.^12–90^

## III. FIBER PRODUCTION VIA MICROFLUIDICS

In addition to the known separation and particle sorting capability^116^ and membrane application,^113–117^ microfluidics has also been employed for fiber production. This new versatile technique has gained increasing interest in recent years as a strong alternative to wet-spinning and electrospinning with higher control over the fiber shape and morphology, offering a gateway for the application of multi-materials to make multicomponent fibers.^83–102^ Although the production speed in microfluidic platforms is typically lower than melt-spinning and solution-spinning, it is a more stable and versatile technology and provides superior control over both the flow of solutions and the characteristics of fibers.^118,119^ Microfluidic spun fibers have attracted considerable attention due to a range of unique advantages they offer, including the control of the fiber size, cost-efficiency, simplified fabrication process, and flexibility, enabling the loading of drugs and biological agents such as proteins, genes, enzymes, and cells in the formed fibers.^36^ On the downside, microfluidic spun fibers typically have lower mechanical properties when compared to fibers made via conventional melt-spinning methods that are subjected to lower drawing ratios, orientation, and crystallinity.

Furthermore, microfluidic devices employed to fabricate such fibers are fabricated through soft lithography, which requires both time and investment in necessary cleanroom facilities initially, despite being a relatively inexpensive method after initial investment.^103–120^ In addition, although the production speed is relatively low, this method is the desired route for the synthesis of advanced and functional fibers for specific purposes, where the throughput is not necessarily essential and microfluidics has the most impact, enabling the production of smaller amounts of higher-value materials. To expand on this, this section highlights the development of fibers having different geometries/shapes and made using various precursor materials through a variety of microfluidic technologies equipped with different solidification methods. In addition, the desired morphologies and fabrication methods for specific applications are described as well.

### A. Conventional materials

The microfluidic spinning of fibrous materials needs the proper selection of materials; for instance, sheath and core solutions pair are essential to achieve a successful *in-situ* cross-linking. Also, the selected materials are responsible for the mechanical properties and pore sizes of the products, which further determine the final application. Meanwhile, biopolymers including the natural [e.g., chitosan, gelatin, collagen, alginate, and polylactic acid (PLA)] and synthesized [e.g., polycaprolactone (PCL), polyvinyl alcohol (PVA), polyethylene glycol (PEG), and polyurethane (PU)] polymers are the most investigated materials for the microfluidic-based fiber production.^38^ A list of different materials used for fiber production and their cross-linking agents/mechanisms, as well as the size, shape/morphology, and applications of the produced fibers, are summarized in Table I.

For instance, hydrogel-based microfibers have been fabricated through microfluidics by coaxial laminar flow^155^ of sample solution (pre-polymer) and sheath fluids (cross-linking agent). Due to the biocompatibility, biodegradability, and mechanical processability of hydrogels, they are suitable candidates for constructing scaffolds.^30^ From the materials mentioned above, alginate is the most frequently used material in the fabrication of cell-laden fibers due to its simple gelation process, biocompatibility, and biodegradability.

**FIG. 4.**
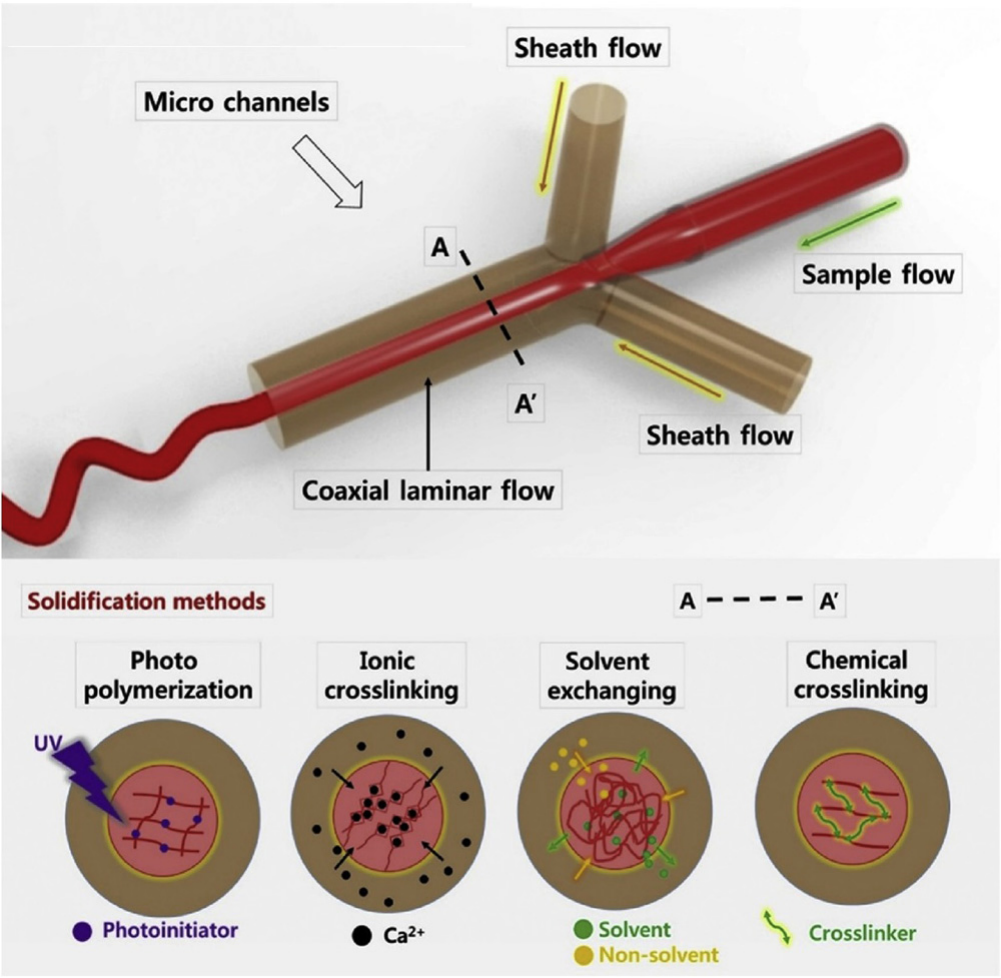
Overview of microfluidic spinning and solidification methods. Reprinted with permission from Cheng *et al*., Biomaterials **114**, 121–143 (2017). Copyright 2017 Elsevier.

Furthermore, several cross-linking or curing methods are employed subsequently after the microfluidic spinning. These methods are categorized into the following four groups: photo-polymerization (UV), ionic cross-linking, solvent exchange, and chemical cross-linking, which are illustrated in Fig. 4.

The wet environment in the microfluidic spinning process paves the way for encapsulating sensitive substances such as cells. Also, this system can be used in the spinning of fibers with diameters ranging from a few micrometers (microfibers) or even on the submicrometer scale (nanofibers) to several hundreds of micrometers. The fibers diameter can be controlled by adjusting the channel dimensions and the flow rates of the sheath and core solutions.

### B. Microfluidic-based fiber production techniques

As mentioned earlier, microfluidics is a powerful technique to fabricate engineered materials like fibers.^14^ As a miniaturized wet-spinning process, microfluidic spinning was developed more than 17 years ago.^38^ The core idea of microfluidic spinning was similar to the mechanism used by silkworms or spiders for fiber production. Jeong *et al.* reported a fiber production method through microfluidic spinning for the first time, noting that the microfluidic spinning can enable the continuous production of microfibers where both the solution flow and final diameter of microfibers are highly controllable.^111^

**FIG. 5.**
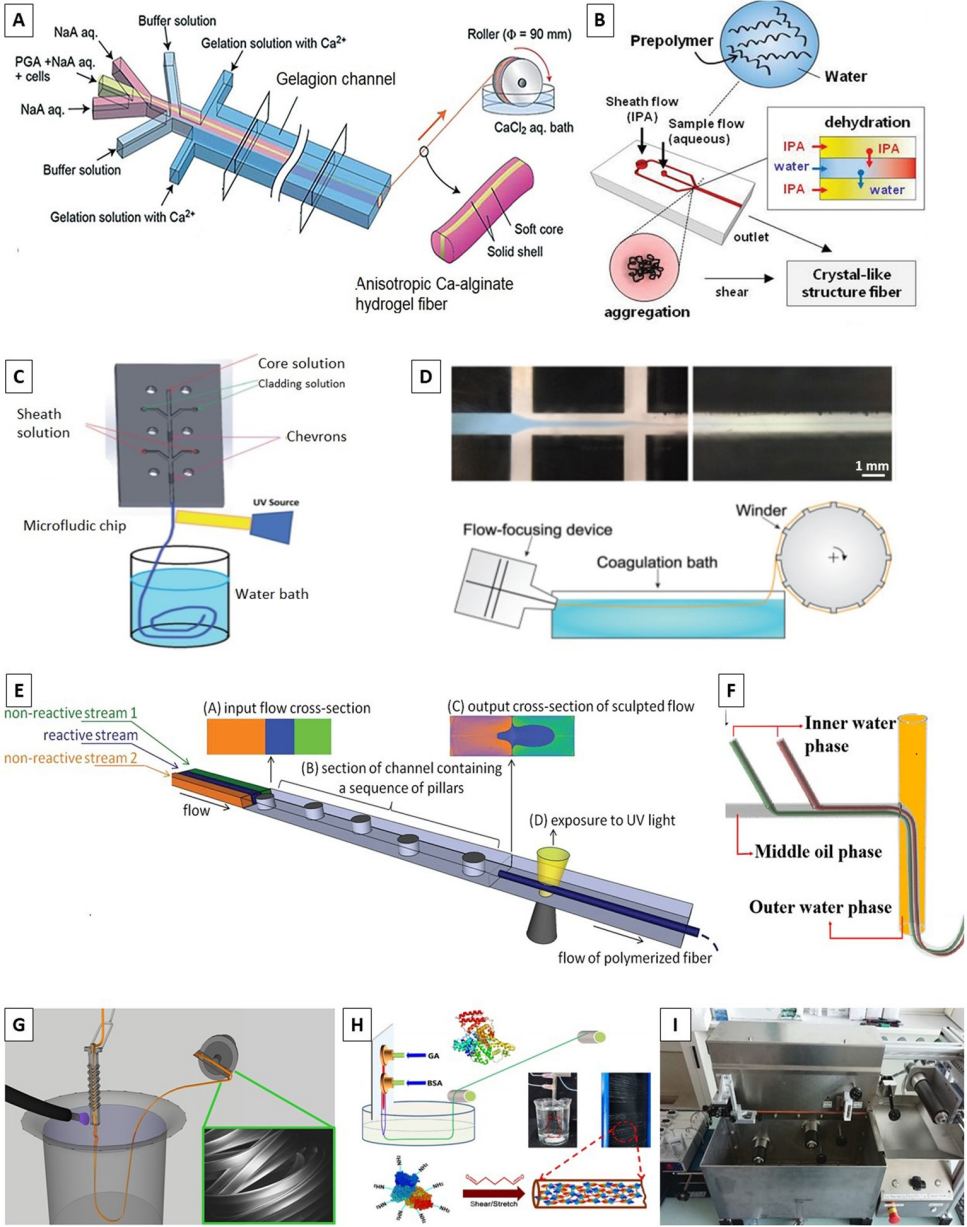
Diverse microfluidic platforms for fiber fabrication: (a) A microfluidic chip made of PDMS with rectangular channels. Reproduced with permission from Jun *et al*., Lab Chip **14**(13), 2145–2160 (2014). Copyright 2014 Royal Society of Chemistry. (b) The silkworm mimicked a microfluidic setup, with isopropyl alcohol (IPA) and alginate as the sheath and core fluids, respectively. Reproduced with permission from Chae *et al*., Adv. Mater. **25**(22), 3071–3078 (2013). Copyright 2013 Wiley-VCH Publishing. (c) A co-flowing and coagulation setup with a UV system for producing elastomeric fibers. Reproduced with permission from Fleischmann *et al.*, Macromol. Chem. Phys. **215**(10), 1004–1011 (2014). Copyright 2014 Wiley-VCH Publishing. (d) A coaxial microfluidic device, including the coagulation bath and winder for fiber production. Reproduced with permission from Nechyporchuk *et al*., Adv. Mater. Technol. **4**(2), 1800557 (2019). Copyright 2019 Wiley-VCH Publishing. (e) Inertial microfluidic process for generating shaped microfibers. Reproduced with permission from Nunes *et al*., Adv. Mater. **26**(22), 3712–3717 (2014). Copyright 2014 Wiley-VCH Publishing. (f) Schematic view of the microfluidic device for spinning editable beaded PLA fibers. Reproduced with permission from Zhang *et al*., J. Colloid Interface Sci. **558**, 115–122 (2020). Copyright 2019 Elsevier. (g) A hydrodynamic focusing microfluidic setup equipped with a UV polymerization device. Reproduced with permission from Boyd *et al*., J. Visualized Exp. **83**, e50958 (2014). Copyright 2014 JoVE. (h) Microfluidic setup for BSA fibers production. Reproduced with permission from He *et al.*, Angew. Chem., Int. Ed. **59**(11), 4344–4348 (2020). Copyright 2020 Wiley-VCH Publishing. (i) Mini wet-spinner. Reproduced with permission from Gursoy *et al*., Polymers **12**(3), 633 (2020). Copyright 2020 Author(s), licensed under a Creative Commons Attribution (CC BY) license.

Fibers with round, hollow, and non-cylindrical cross sections (e.g., flat, ribbon, grooved, and belts) have been produced using PDMS microfluidic devices with rectangular channels^36^ [Fig. 5(a)]. Also, some complex microfluidic systems have been employed to fabricate multi-component fibrous materials.^49^ Recently, different methods have reported the precise fabrication of microfibers with controllable tubular, spindle-knot-like, and peapod-like internals.^2–49^ The tubular fibers allow efficient encapsulation of cells and phase change materials in high content. The peapod-like microfibers with separate oil cores can serve as multicomponent systems for the encapsulation of multiple drugs. The microfibers with magnetic spindle-knot-like internals can be assembled into spider-web-like structures for water collection. On a similar note, Shi *et al.* developed a novel microfluidic technique to produce bioinspired microfibers with hourglass knots by the integration of non-solvent-induced phase separation (NIPS).^54^ Post-treatment of the partially gelled spindle-fibers in ethanol causes the encapsulated oil cores to leak from the knots, which will result in changing the morphology of the fibers into an hourglass shape. Also, Brown *et al.* reported a microfluidic approach to produce metal-organic frameworks (MOFs) through a two-solvent interfacial method for positional control over membranes in polymeric hollow fibers for the continuous formation of the desired membrane (i.e., made of zeolitic imidazolate framework, ZIF-8).^13^

Alginate, as mentioned above, is one of the most suitable materials for biomedical applications. Therefore, there have been remarkable efforts made on the production of alginate fibers through microfluidics. For instance, Shin *et al.* reported a continuous microfluidic technique to produce calcium alginate fibers, using a core fluid (sodium alginate solution) and a sheath fluid (CaCl_2_ solution) to generate the final fibers by the coagulation process.^47^ In the same vein, Chae *et al.* simulated the silkworm spinning process to produce microscale alginate fibers through a microfluidic channel that is 100 *μ*m in diameter.^41^ As depicted in Fig. 5(b), they used sodium alginate (1% w/v) as the core solution and isopropyl alcohol (IPA) as the sheath solution. Their experimental trials with different core–sheath solution concentrations and flow rates resulted in the formation of microfibers with highly ordered and crystalline structures, having various morphologies. In another work by Cuadros *et al.*, calcium alginate fibers were produced with uniform diameters employing a microfluidic strategy.^112^ The effect of the concentrations of the sodium alginate and calcium chloride (CaCl_2_) solutions was evaluated on the mechanical properties of fibers. It was shown that the tensile stress of fibers was increased with the Ca^2+^ concentration increment, up to a certain point of 1.4%. Also, Liu *et al.* developed a coaxial microfluidic device to produce helical alginate hydrogel microfibers with flexible shapes by adjusting the flow rates of the core and sheath streams.^50^ They evaluated the effect of the guluronic (G-block)/epimer mannuronic (M-block) residues (G/M) ratio of the alginates on the microfiber coiling phenomenon. On the same topic, Peng *et al.* developed a microfluidic spinning platform coupled with a free radical polymerization system to fabricate graphene oxide/polyacrylamide/sodium alginate hydrogel fibers.^64^ The produced fibers showed high mechanical, stretching, and electro-responsive properties, making them a potential candidate for application as an artificial muscle actuator. The electro-response rate of the fibers can be improved by graphene oxide content increment, *N,N*-methylenebisacrylamide (BIS) content, and fiber diameter decrement. Chaurasia and Sajjadi developed a buoyancy-assisted microfluidic device to fabricate air-filled alginate microfibers with tunable encapsulation and fiber morphology via a coaxial flow of an aqueous sodium alginate solution enveloping an air phase, injected into a quiescent aqueous CaCl_2_ solution.^53^ Using a different type of microfluidic setup, i.e., a flow-focusing microfluidic chip, Martino *et al.* developed a microfluidic approach to segment an alginate precursor solution and generate alginate fibers in various lengths, ranging from 100 *μ*m up to 1 mm.^86^

Besides alginate, other bio-based materials have also been used for microfluidic fiber production. For instance, Rodríguez-San-Miguel *et al.* developed a microfluidic setup for the reaction between 1,3,5-tris(4-aminophenyl) benzene and 1,3,5 benzenetricarbaldehyde solutions in acetic acid to produce, for the first time, highly crystalline and porous fibers of covalent organic framework polymers consisting of micro-fibrillar structures.^10^ In another research, Morimoto *et al.* used a 3D printed module along with the coaxial microfluidic device to produce hydrogel fibers.^109^ They also demonstrated the capability of their setup to fabricate a multi-layered cell-laden fiber through the gelation of cell-laden collagen solutions in a multi-layered laminar flow condition. Pullagura and Gundabala presented a microfluidic method for fiber production, with the capability to control the fiber size and the extent of coiling of the generated fiber for manufacturing non-woven and single polyethylene oxide (PEO) fibers.^44^ Moreover, Li *et al.* produced aligned hierarchical-structured silk fibers by integration of “bottom–up” and “top–down” strategies.^81^ They dispersed and assembled silk nanofibers (SNFs) in formic acid and spun them into aligned and structural fibers via a bioinspired microfluidic setup. Fleischmann *et al.* developed a microfluidic co-flowing setup assisted with a UV cross-linking device [Fig. 5(c)] to generate elastomeric thermoresponsive fibers using liquid crystalline polymers (i.e., polyacrylate).^71^

**FIG. 6.**
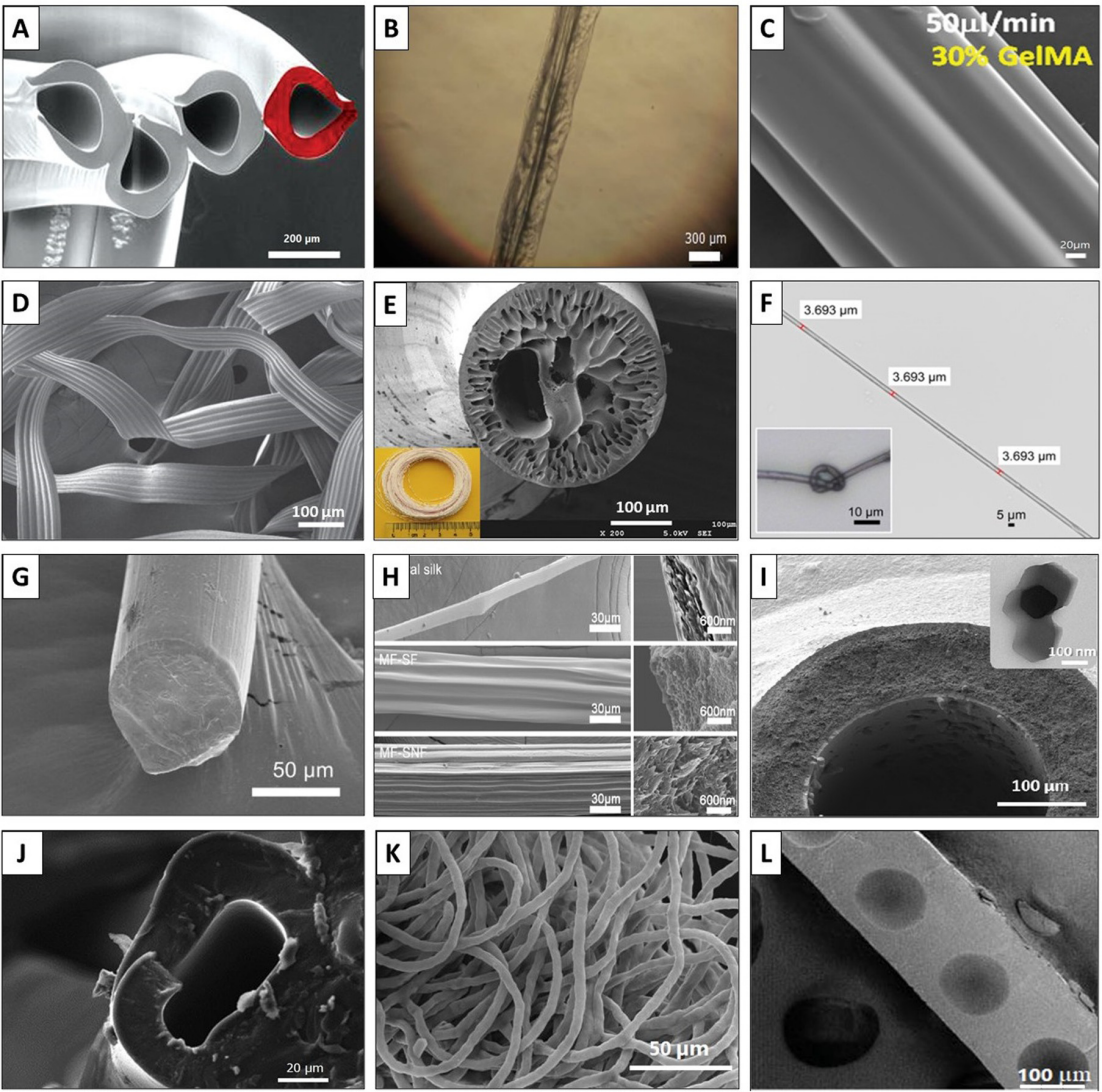
Microscopic images of microfluidic spun fibers: (a) The cross section of the microfluidic spun shaped-fiber using interfacial microfluidics. Reproduced with permission from Nunes *et al*., Adv. Mater. **26**(22), 3712–3717 (2014). Copyright 2014 Wiley-VCH Publishing. (b) Multicomponent hydrogel fibers without (−CHT) and with chitosan (+CHT). Reproduced with permission from Costa-Almeida *et al.*, ACS Biomater. Sci. Eng. **3**(7), 1322–1331 (2017). Copyright 2017 American Chemical Society. (c) Field emission-scanning electron microscopy (FE-SEM) image of a micro-structured GelMA fiber. Reproduced with permission from Shi *et al*., Adv. Funct. Mater. **25**(15), 2250–2259 (2015). Copyright 2015 Wiley-VCH Publishing. (d) SEM image of flat fibers with microgrooved patterns. Reproduced with permission from Kang *et al*., Adv. Mater. **24**(31), 4271–4277 (2012). Copyright 2012 Wiley-VCH Publishing. (e) Optical and electron microscopy images of a PVB fiber. Reproduced with permission from Wen *et al*., Appl. Therm. Eng. **87**, 471–480 (2015). Copyright 2015 Elsevier. (f) Optical microscopy image of a microfluidic spun collagen microfiber. Reproduced with permission from Haynl *et al*., Nano Lett. **16**(9), 5917–5922 (2016). Copyright 2016 American Chemical Society. (g) SEM image of an hourglass-shaped microfiber. Reproduced with permission from Shi *et al*., ACS Appl. Mater. Interfaces **12**(26), 29747–29756 (2020). Copyright 2020 American Chemical Society. (h) Cross-sectional and bilateral views of natural and microfluidic-fabricated (MF) silk fibers (SF) and nanofibers (SNF). Reproduced with permission from Li *et al*., ACS Biomater. Sci. Eng. **6**(5), 2847–2854 (2020). Copyright 2020 American Chemical Society. (I) Microfluidic spun ZIF hollow membrane with crystals indicated on the wall. Reproduced with permission from Cacho-Bailo *et al*., J. Membr. Sci. **476**, 277–285 (2015). Copyright 2015 Elsevier. (j) SEM image of the cross section of a microfluidic-based PEGDA hollow micro-vessel. Reproduced with permission from Aykar *et al*., RSC Adv. 10(7), 4095–4102 (2020). Copyright 2020 Royal Society of Chemistry. (k) SEM image of microfluidic spun PEO fibers. Reproduced with permission from Pullagura and Gundabala, Langmuir **36**(5), 1227–1234 (2020). Copyright 2020 American Chemical Society. (l) An air-dried microfluidic spun peapod-like chitosan microfiber. Reproduced with permission from He *et al*., RSC Adv. **5**(2), 928–936 (2015). Copyright 2015 Royal Society of Chemistry.

Furthermore, Haynl *et al.* presented a microfluidic strategy to produce collagen microfibers yielding the formation of microfibers with diameters as small as 3 *μ*m.^45^ Polarized Fourier transforms infrared spectroscopy (FTIR) investigations confirmed that the induced fibril orientation along the microfiber axis gives rise to the outstanding mechanical stability of the produced fibers exceeding that of the natural tendon fibers and collagen fibers. They concluded that these fibers are good candidates for biomedical applications, especially peripheral nerve repair and regeneration. In another work, Nechyporchuk *et al.* developed a double sheath flow-focusing microfluidic system [Fig. 5(d)] for the spinning of mechanically strong fibers from cellulose nanocrystals (CNCs) and nanofibrils (CNFs).^11^ Nunes *et al.* reported a microfluidic methodology [Fig. 5(e)] for the synthesis of pre-designed shaped polymeric fibers using a software-enabled inertial.^7^ In this technique, fluid streams can be sculpted into designed shapes in a microchannel with a sequence of pillars. The synthesized shaped fibers are shown in Fig. 6(a). Also, Zhang *et al.* used a microfluidic approach for the spinning of editable polychromatic polylactic acid (PLA) fibers [Fig. 5(f)].^35^ Microfluidic spun polychromatic PLA fibers demonstrated the capability to deliver coded information through editable chromatic behavior.

Using a different type of setup, Wen *et al.* developed a microfluidic system to fabricate core–shell phase change microfibers with high paraffin Rubitherm27 (RT27) content.^55^ Microfluidic spun fibers were composed of the poly(vinyl butyral) (PVB) sheath and paraffin core, protected from leaking during the phase change process. The amount of paraffin in the fabricated microfibers was controlled by adjusting the inner flow rate with the maximum content of 70%, resulting in the maximum crystallization enthalpy and melting enthalpy for an excellent thermal performance. Employing a similar approach, Zhang *et al.* produced core–sheath composite fibers with the phase change and enhanced thermal conductive performance.^60^ These fibers consist of an RT27 core and PVB sheath reinforced with aluminum oxide nanoparticles (Al_2_O_3_ NPs). This approach resulted in the successful production of phase change microfibers with heat conductive properties and fast thermal regulation properties. Ligler's group developed a co-flow microfluidic strategy assisted with a rapid UV-polymerization system [Fig. 5(g)] to produce pre-designed polymeric fibers with the desired size and shapes.^8–156^ They also made Thiol click fibers, where the core streams of thiolene and thiolyne prepolymer solutions were guided using a phase-matched sheath stream through microfluidic channels with grooved walls to form the desired shape.^92–156^ Thiol click reaction was initiated by the UV illumination to lock-in the designed cross section and size for the fibers. Also, they functionalized the surface of the fiber with a covalently attached ligand.^92–156^ Shields *et al.* used the strategy developed by the Ligler's group^92–156^ to generate a continuous flow for the fabrication of long fibers with desired shapes through hydrodynamic shear forces, molecular-scale assembly, hydrodynamic focusing, and advection driven by grooves in the channel walls.^101^ Similarly, Thangawng *et al.* employed a core–sheath flow microfluidic setup, assisted with a UV polymerization device to fabricate polymethyl methacrylate (PMMA) fibers with desired shapes and sizes.^93,94^ They reported the hydrodynamic focusing as the main parameter affecting the fiber shape, while the sheath/core solutions flow rate ratio is critical for adjusting the fiber diameter.^94^ Round PMMA fibers with diameters down to 300 nm were produced by adjusting the flow rate ratio between the sheath and core solutions via a five-diagonal grooved device. Ribbon-shaped fibers with a submicrometer thickness were also fabricated using a seven-chevron/five-diagonal grooved combination device.^93^ Also, Aykar *et al.* developed a microfluidic platform assisted with an attached photopolymerization device for the fabrication of poly(ethylene glycol diacrylate) (PEGDA)-based hollow fibers as the self-standing micro-vessels with inner diameters ranging from 15 to 73 *μ*m and biocompatibility/cytocompatibility.^95^

He *et al.* demonstrated the fabrication of robust protein fibers using bovine serum albumin (BSA) via a microfluidic technique [Fig. 5(h)].^52^ In another work, Meng *et al.* developed a microfluidic system to fabricate and design ordered porous and anisotropic core–sheath fibers based on nickel oxide arrays (sheath) and graphene nanomaterials (core).^114^ Honaker *et al.* produced a bicomponent fiber consisting of liquid crystal (core) and polyisoprene rubber (sheath) through a laboratory-scale microfluidic.^51^ The developed fibers are stretchable, maintaining their core integrity under substantial strain; the unique feature that makes them a potential candidate for the application in tensile sensors and soft robotic actuators.

In a different work, Gursoy *et al.* developed a facile technique using hypodermic needle arrays within a silicone elastomer matrix to customize a microfluidic spinning system [Fig. 5(i)], where the microfluidic spinnerets display coaxially aligned channels.^103^ The developed setup exhibited the capability of operating under laminar flow regimes and achieving precise 3D hydrodynamic flow focusing. They exemplified the performance of the developed microfluidic setup by producing fibers with desired morphologies using commercial polyurethane by varying the degree of flow focusing.

### C. Microfluidic spun fibers

As described in Sec. III B, various fibers with different morphologies can be produced using polymeric materials by microfluidic spinning methods. Processing parameters and the materials (polymer and fluids) can affect the characteristics of the produced microfibers, e.g., the cross section, strength, degradability, porosity, and morphology, among others. These fibers are regarded as prototype products for a range of specific applications, including but not limited to cell culture, tissue engineering, drug delivery, optical sensors, and micro-electronics, which are discussed in Sec. IV.

For instance, Nunes *et al.* produced microfluidic spun shaped fibers shown in Fig. 6(a) using interfacial microfluidics.^7^ Costa-Almeida *et al.* combined the polyelectrolyte complexation with microfluidics to generate hydrogel fibers with a fibril-like structure.^106^ They mixed chondroitin sulfate (MA-CS) or methacrylate hyaluronic acid (MA-HA) with alginate, which is negatively charged, combined with positively charged chitosan, and separately injected into a microfluidic device to obtain multicomponent hydrogel fibers. These microfluidic spun hydrogel fibers exhibited smaller fibrils aligned in parallel whenever the chitosan was present [a microscopic image of one of these fibers is depicted in Fig. 6(b)]. According to the work by Meng *et al.*, graphene-doped core–shell fibers were synthesized through a homogeneous reaction within a microfluidic device,^114^ where the core maintains a uniformly anisotropic porous structure while the nickel oxide sheath remains aligned. The produced fibers present an ultrahigh energy density and high specific capacity. Nguyen *et al.* produced hollow alginate fibers in a triple-flow PDMS-based microfluidic device.^37^ The alginate hollow fibers showed unique characteristics, like flexibility, while exhibiting robust mechanical strength, biocompatibility, and permeability, making them a suitable candidate for scaffolds in terms of the attachment, culture, and proliferation of human umbilical vein endothelial cells (HUVECs) to eventually fabricate an artificial blood vessel.

Microgroove patterning is an interesting area in the microfluidic spinning of fibers, employed to fabricate fibers from GelMA^102^ and alginate,^83^ as illustrated in Figs. 6(c) and 6(d), respectively. PVB/paraffin core–shell fibers produced through phase change solidification are shown in Fig. 6(e).^55^ As shown in Fig. 6(f), Haynl *et al.* produced a microfluidic spun collagen fiber 3 *μ*m in diameter, which is fine enough for making a knot.^45^ Furthermore, a section of hourglass fibers produced by Shi *et al.* is presented in Fig. 6(g). The brittle fractured cross section of the fiber shows a round shape that is about 50 *μ*m in diameter.^54^ The microfluidic spun hierarchical-structured silk fibers and nanofibers by Li *et al.* are compared with natural silk fiber in Fig. 6(h).^53^ Hollow fibers are also one of the important classes of spun fibers for membrane applications [Fig. 6(i)],^113^ micro-vessels [Fig. 6(j)],^95^ and micro-pipes,^157,158^ among others. Finally, Figs. 6(k)–6(l) show microfluidic spun PEO fibers^44^ and peapod-like chitosan fibers,^74^ respectively. These fibers exhibit profound flexibility and strength, making them a potential candidate for many biomedical applications.

As shown in Fig. 6, fibers with a variety of shapes and geometries, including solid, core–shell, hollow, parallel, porous, grooved, and flat fibers with different structures and morphologies, could be produced based on the design of the microfluidic devices and the material(s) selected.^38^

### D. Treatment and analysis

To enhance their functionality, some microfluidic spun fibers need to be treated before the final application. Treatments can be chemical such as the alkaline reduction and UV reaction or mechanical such as the sandblast and etch. For instance, Boyd *et al.* treated the surface of the fabricated fibers with a covalently attached ligand.^92–156^ Park *et al.* prepared a bundle of the microfluidic spun fibers to develop an ophthalmology suture that concluded in a porcine eye with a smoother post-operative surface compared with a nylon suture.^40^ They developed a method to increase the mechanical performance of fibrous biomaterials for different applications such as medical purposes.

As mentioned earlier, microfluidics is a suitable method for the encapsulation of cells within polymeric fibers like alginate for biomedical applications such as the fabrication of artificial blood vessels, muscle, and tendon, or broadly in tissue engineering. Fibers need a high surface area and low contact angle for an effective attachment to the biomaterials, a clear example of where the surface treatment comes into play. For instance, Shi *et al.* used the microfluidic technologies along with a post-treatment mechanism to fabricate photo-crosslinkable methacrylamide-modified cell-responsive gelatin (GelMA) fibers with exquisite structured surfaces.^102^ GelMA fibers promoted the viability of encapsulated cells compared with similar grooved alginate fibers prepared as a control sample. Moreover, the shaped cross-sectioning or microgrooves are among the best routes for the surface increment.^102–119^ In this regard, Kang *et al.* produced flat alginate fibers with grooves through a microfluidic-based method for the neural cells culture.^83^ They constructed the coaxial grooved slit channel by aligning and bonding two grooved PDMS channels using the oxygen plasma treatment. Of particular note, plasma is a relatively new technique for the surface treatment (e.g., hydrophilic or hydrophobic surface characteristic) employed in different fields, including microfluidic-based fabrication of fibers for typical applications.^159^ Therefore, surface treatments can result in changing some of the key characteristics of microfluidic spun fibers.

In addition to performing the conventional analysis of the morphological, physical, and mechanical properties of the microfibers produced via experimental trials, the coagulation, diffusion, gelation, drawing, orientation, and crystallization of the microfluidic spun fibers have also been studied using computational fluid dynamics (CFD) analysis and theoretical approaches.^40–162^ To discuss a couple of examples, Park *et al.* demonstrated that a microfluidic device for conventional fiber spinning has the potential to control the mechanical performance of a single microfiber.^40^ They developed a mathematical equation for this capability, explaining the mechanical property control of the single poly(l-lactic-co-ε-caprolactone) (PLCL) fibers. They suggested a simple equation for the relationship between the length density (ρ_l_) and initial Young's modulus (iYm). iYm was determined from the slope between 0% and 20% of the strain in the stress–strain curve. They provided the following equation:

$$\;\rho_{l}\propto \frac{Q_{\mathrm{PLCL},k}\times A_{k}}{Q_{\mathrm{MeOH},k}},$$

where k depends on the condition, Q_PLCL,k_ is the core solution flow rate, Q_MeOH,k_ is the sheath solution flow rate, and A_k_ is the cross-sectional area of the fiber. In another work, Bonhomme *et al.* conducted a theoretical analysis on alginate fiber formation in a microfluidic spinning setup.^91^ They explored the conditions under which fibers can be fabricated using a microfluidic device with the gelation of the alginate with a calcium salt. Put simply, they investigated the effect of the control parameters such as the salt concentration, residence time, and size of the polymeric jet on the fiber production.

## IV. APPLICATIONS OF MICROFLUIDIC SPUN FIBERS

Microfluidic spun micro-/nano-fibers have emerged as a class of promising materials for biomedical and environmental applications on account of their interesting physical and chemical properties, including high surface-area-to-volume ratio, effective heat transfer, biocompatibility, and reaction rate. As presented in Table I, microfluidic systems enable the production of different polymeric fibers with various morphologies and constructions, including solid, hollow, core–shell, multi-layered, grooved, heterogeneous, and helical. These materials have mainly been developed to feature multifunctionality and applicability to new biosensors, wearables in medicine, tissue engineering, drug delivery, and optical sensors,^49^ which are discussed in this section.

### A. Micro-/nano-fiber-based sensors

**FIG. 7.**
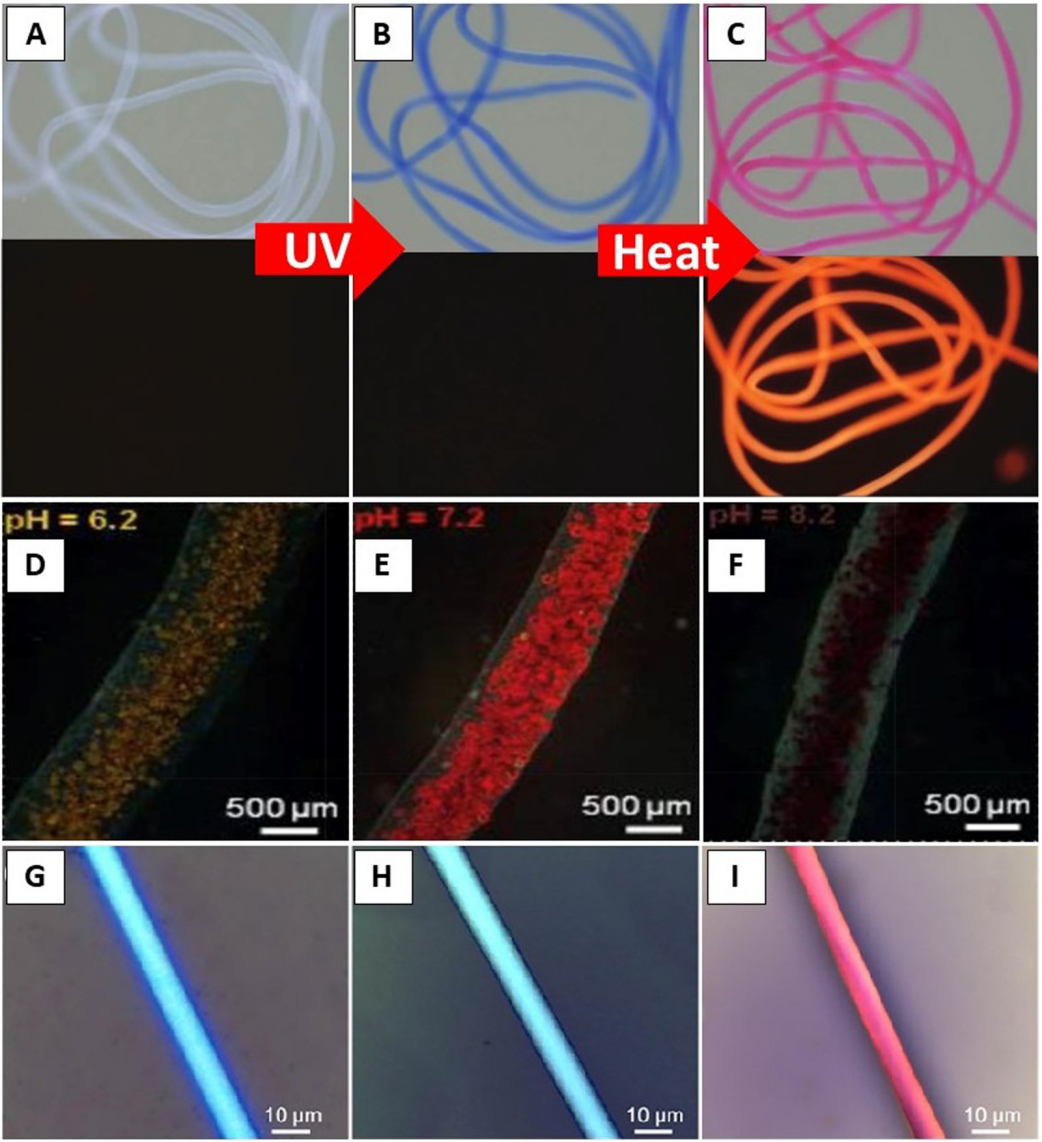
(a)–(c) Optical and fluorescent images of polydiacetylene fibers before (a) and after UV (b) and heat treatments (c). Reprinted with permission from Yoo *et al*., Macromol. Rapid Commun. **33**(15), 1256–1261 (2012). Copyright 2012 Wiley-VCH. [(d)–(f)] Images of pH-sensitive wound dressing fibers with solutions having different pH: 6.2 (d), 7.2 (e), and 8.2 (f). Reprinted with permission from Tamayol *et al*., Adv. Healthcare Mater. **5**(6), 711–719 (2016). Copyright 2017 Wiley-VCH. (g)–(i) Microscope images of the crystal photonic fibers with blue (g), green (h), and red (i) structural colors. Reprinted with permission from Li *et al.*, Mater. Lett. **242**, 179–182 (2019). Copyright 2019 Elsevier.

Micro-/nano-fiber-based materials have been explored as a matrix for biomedical and environmental applications.^163–165^ They are usually spun fibers loaded with indicators capable of reacting in contact with external stimuli. For instance, Yoo *et al.* developed fluorescent polydiacetylene-embedded alginate microfibers to determine metal ion levels in the aqueous solutions [Figs. 7(a)–7(c)].^166^ When employed as a sensor, these fibers have unique characteristics because they can undergo a blue-to-red color change and non-fluorescence-to-fluorescence transition in response to environmental stimuli.^166^ These researchers used a microfluidic technique to produce an insoluble hydrogel from calcium ions before assembling diacetylene-surfactant complexes in the calcium alginate fibers. UV irradiation of the fibers produced blue color diacetylene, and the formation of a conjugated polymer was confirmed by heat-induced phase transition and Raman spectroscopy.^166^ Strikingly, these fibers were also proved to be capable of detecting different types of cyclodextrin concentrations, while cyclodextrin is the Food and Drug Administration (FDA) approved solubilizing agent to improve the drug delivery efficiency in the human body.^167^

In another work, Tamayol *et al.* developed a pH sensor by storing an indicator on the hybrid hydrogel microfibers for the point-of-care detection of pH on an epidermal wound, as shown in Figs. 7(d)–7(f).^168^ The pH of the human skin surface is normally between 4.2 and 5.6 and decreases with the lapse of epithelialization, the characteristic that the developed sensor leverage to screen the wound healing process.^169^ In addition, there is a colorimetric fiber-based humidity sensor developed by Chen's group using a homogeneous mixture of viscous polyvinylpyrrolidone and monodisperse SiO_2_ particles in a microfluidic device. This sensor could produce colorimetric signals over almost the entire visible spectrum in response to the environmental humidity. Photonic crystal structures can also be constructed by removing polyvinylpyrrolidone from the SiO_2_ particles through calcination. The color of the resulting crystal fibers can be tuned by changing the sizes of SiO_2_ particles [Figs. 7(g)–7(i)].^170^

Multicomponent fibers have further been fabricated using microfluidic technologies for the detection of multiple target molecules in parallel. These types of fibers can be used to improve the analysis accuracy and information capacity for different applications. To use this concept, Cho *et al.* synthesized PEG-DA-based microfibers to perform multiplex immunological assays.^171^ The fibers were first functionalized with human and rabbit antibodies before applying analyte samples containing the fluorescein isothiocyanate and tetramethyl rhodamine isothiocyanate conjugated with anti-human and anti-rabbit antibodies, respectively. The detection limit was determined to be as low as 0.01 pg/ml for both antigens.^171^ Leveraging a microfluidic spinning system, Nakajima *et al.* also developed a microfiber-based pH/temperature dual sensor, employing a stimuli-responsive hydrogel, poly(*N*-isopropylacrylamide) (pNIPAM), which can repeatability shrink and swell in response to the temperature and/or pH change.^172^ These sensors can be used for a variety of applications, including drug releasing systems, modification of cell-culture surfaces, and biochemical sensing. They also demonstrated that the response rate of these sensors is directly related to the diameter of the fibers. As demonstrated by research, microfiber-based materials have paved the way for developing many novel sensors in translational research areas.

### B. Micro-/nano-fiber-based wearable electronic devices

Wearable biosensors recently received extensive attention due to the real-time self-measurement of the physical status as well as the screening of physiological parameters. These sensors show excellent potential in monitoring the metabolic status of the body, diagnosis, and treatment.^173^

**FIG. 8.**
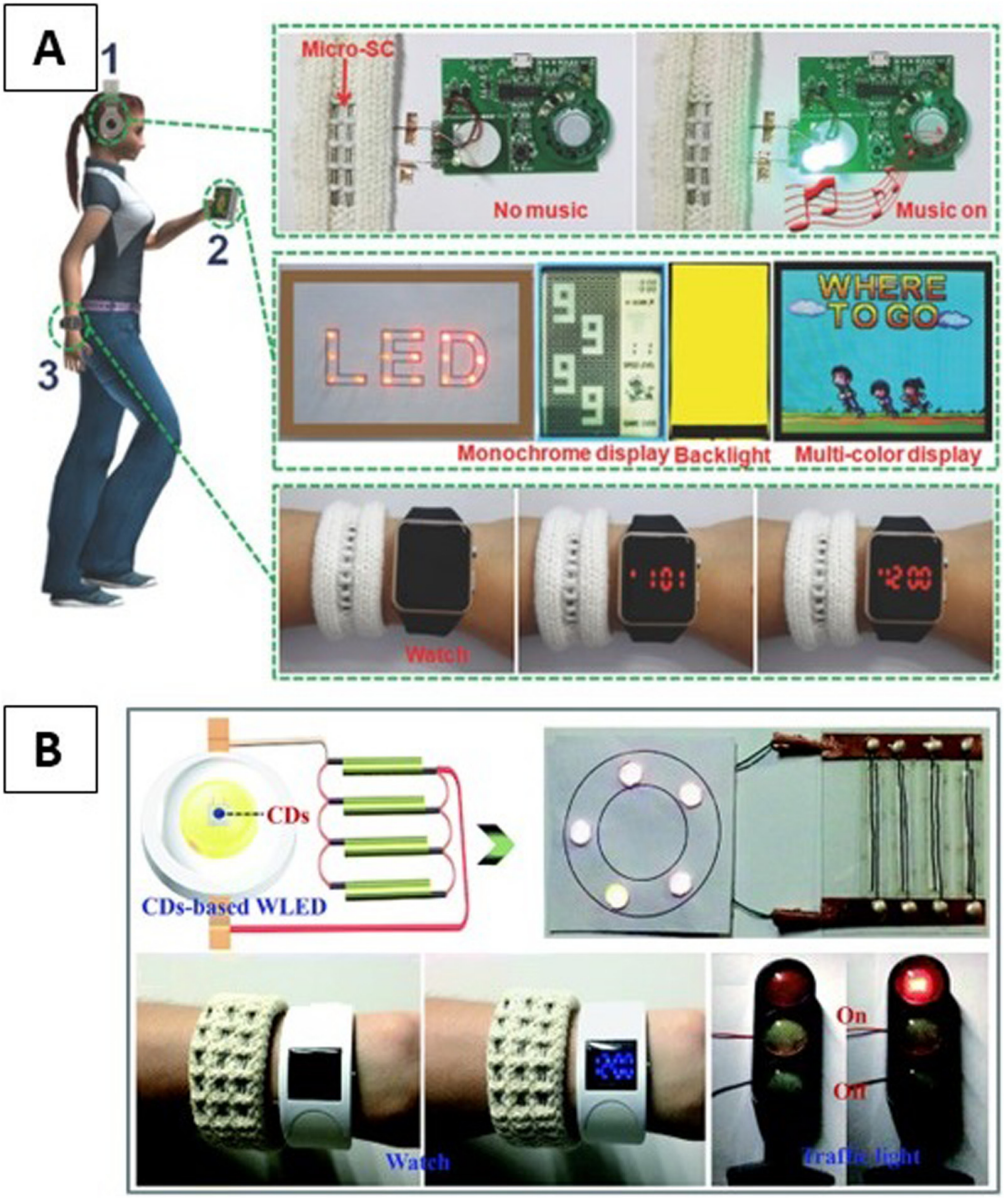
(a) Schematic illustration and photographs of the micro-supercapacitor integrated into the woven fabric and flexible substrates powering various electronics such as audio, LEDs, monochrome display, backlight, multicolor display, and watch. Reprinted with permission from Wu *et al.*, Adv. Funct. Mater. **27**(36), 1702493 (2017). Copyright 2017 Wiley-VCH Publishing. (b) Images of micro SCs embedded into a CD-based WLEDs, a smartwatch, and traffic lights. Reprinted with permission from Li *et al*., J. Mater. Chem. A **6**(29), 14112–14119 (2018). Copyright 2018 Royal Society of Chemistry.

Microfluidic spinning technology has demonstrated techniques for the large-scale production of micro-/nano-fiber-based supercapacitors with outstanding mechanical properties and superior electrochemical performance. Flexibility, biocompatibility, embeddability, durability, high power density, and fast charge/discharge rate of microfiber-based supercapacitors make them prime candidates for fabricating wearable biosensors. The combination of the nanocarbon with electrochemically active materials has been employed to fabricate microfibers, which can be used to develop flexible supercapacitors with high energy storage capacity for practical applications. As an example, Xu *et al.* developed an approach for the continuous production of graphene fibers with sandwich structures using a three-phase microfluidic spinneret.^174^ They first prepared a solution by dissolving the sodium alginate and polyvinyl alcohol in water and stirring the solution overnight before use. The solution was then injected into the middle channel of a microfluidic device while graphene oxide was injected into the side microchannels as the sheath stream *via* syringe pumps. Subsequently, both solutions were simultaneously extruded out of the outlet into the coagulation bath to obtain a solidified hybrid fiber. Finally, the graphene oxide of the side microchannels was reduced under hydrazine vapor to create a supercapacitor.^174^ The resulting fiber-based supercapacitor demonstrated good electrochemical properties and possessed high flexibility and mechanical performances suitable for use in wearable sensors. In another study, Wu *et al.* fabricated homogeneous nitrogen-doped porous graphene fibers as micro-supercapacitors using graphene oxide and urea through a microfluidic-directed strategy [Fig. 8(a)].^175^ These micro-supercapacitors could be embedded into wearable products by demonstrating an ultra-large specific capacitance of 1132 mF/cm^2^ and a high energy density of 95.7 *μ*Wh/cm^2^ as power electronics. In a follow-up work from the same group, they improved the design using a unique dot sheet structure fabricated from carbon dots (CDs) and graphene [Fig. 8(b)].^176^ The new design demonstrated a larger specific surface area, more ionic channels, and excellent mechanical strength, resulting in a 22.1% enhancement of capacitance. Furthermore, Pan *et al.* produced highly oriented GO-molybdenum disulfide (MoS_2_)/cellulose nanocrystal microfibers using a purpose-designed microfluidic chip. The electrical conductivity of the microfibers was determined as ∼3 × 10^4^ S/m leading to a high power density in an aqueous electrolyte.^177^

### C. Fiber-based scaffolds for tissue engineering

Tissue engineering is an interdisciplinary field of life science that employs both engineering and biological principles to regenerate tissues and organs.^178^ One of the key elements used in tissue engineering approaches is the scaffold, which supports and directs the growth of the cells, and the successful fabrication of functional scaffolds requires biodegradable and biocompatible materials. Furthermore, cell proliferation, differentiation, and tissue formation are affected by the geometries, morphologies, and mechanical properties of scaffolds. To address these requirements, the electrospinning technology was developed to fabricate nonwoven fiber-based scaffolds; however, this technology requires costly equipment, suffers from difficulties in the encapsulation of cells, and lacks control over the fabrication of complex structures. By contrast, microfluidic technologies have been employed to manipulate the geometry, composition, and structure of fiber-based scaffolds by constructing, better handling, and assembling building units using conventional microfluidic devices. The resulting scaffolds could be used to fabricate complex human tissues and organ-on-a-chip. Using a microfluidic spinning device, Yamada *et al.* developed the anisotropic Ba-alginate microfibers with hepatocytes in the middle sandwiched by 3T3 cells. They showed that the cell-incorporated systems maintained high hepatocyte viability (∼80%) over a month.^179^

Jia *et al.* also reported the fabrication of complex cell-laden helical structures using hollow hydrogel microfibers by tuning the flow rates or modifying the geometry of a conventional microfluidic device.^72^ These microfibers could mimic the structural characteristics of helical blood vessels and generate swirling blood flow on a chip. These types of hydrogel-based helical microstructures have potential applications in areas such as blood vessel tissue engineering, organ-on-a-chip, drug screening, and biological actuators. After cultivating human umbilical cord vein endothelial cells (HUVECs) for seven days, the cells were shown to completely adhere to the inner layer of the hollow helical fibers, and the proliferation occurred.^72^ In another study, Wei *et al.* also developed spatial cell-laden double-layered hollow microfibers with the encapsulation of HUVECs and osteoblast-like MG63 cells.^48^ The biomimetically engineered osteon microfibers with reinforced vasculogenic and osteogenic expression were assembled into a sophisticated tissue-like structure. Employing a microfluidic device, Kobayashi *et al*. developed stripe-patterned heterogeneous hydrogel sheets with alternant embeddedness of hepatoma cells (HepG2) and fibroblasts (Swiss 3T3) to mimic the heterotypic hepatic cord structures and stimulate hepatic functions.^180^ Other researchers also fabricated artificial tissues by assembling fiber-like constructs with varying measures. For instance, Lee *et al.* demonstrated the fabrication of 3D artificial micro-vessels based on HIVE-78 cell-encapsulated hollow alginate microfibers. They embedded the cell-laden fibers into agar-gelatin-fibronectin hydrogels and co-cultured them with the muscle cells (HIVS-125).^69^

**FIG. 9.**
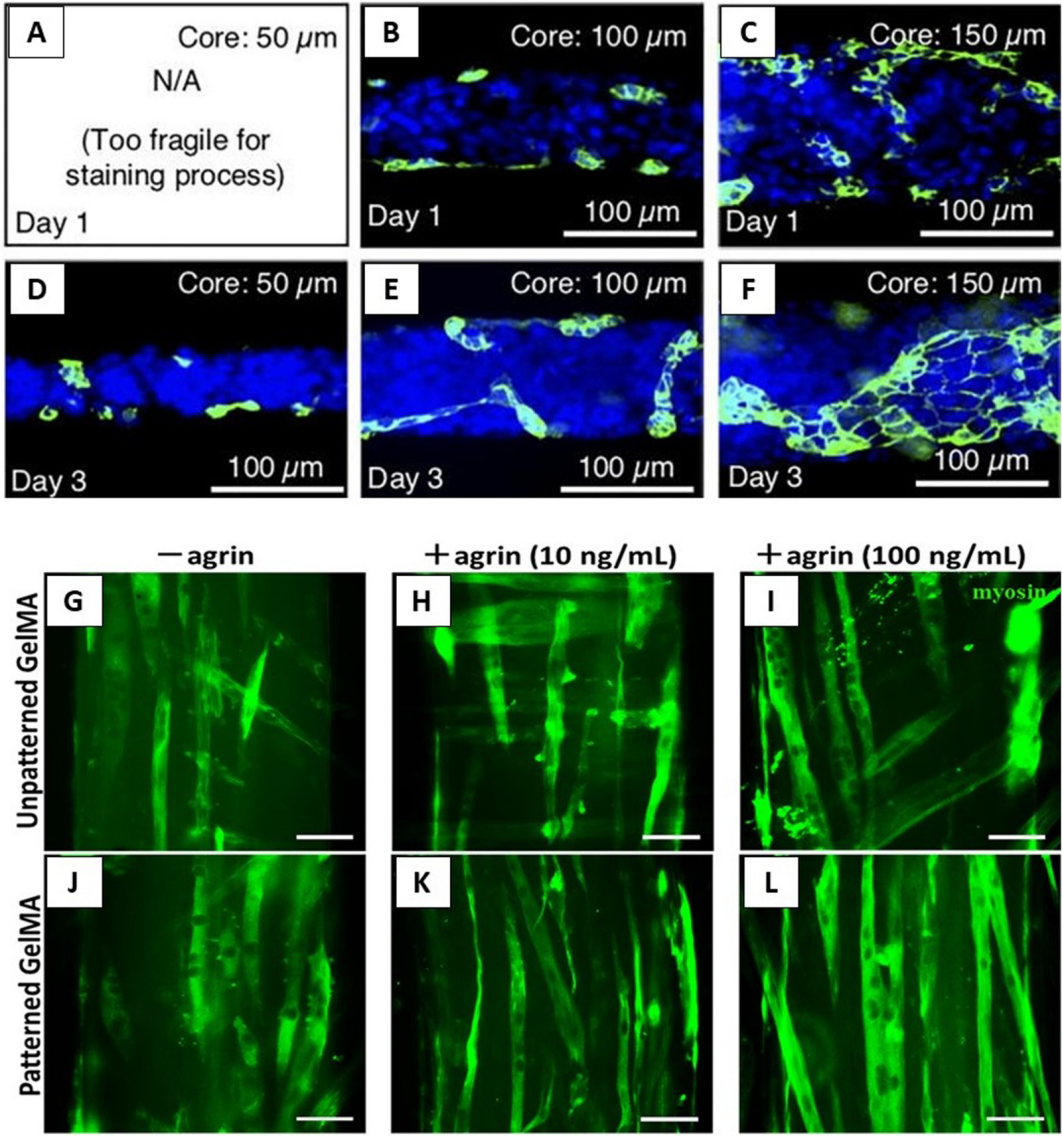
(a)–(f) Confocal fluorescent microscopic images of HUVEC networks with different core diameters in the microfiber-shaped tissue from day 1 to day 3 (the cells and nuclei of cells are stained green and blue, respectively). Reprinted with permission from Kurashina *et al*., APL Bioeng 3(4), 046101 (2019). Copyright 2019 American Institute of Physics. [(g)–(l)] Confocal fluorescent microscopic images of myotubes formed on micropatterned and unpatterned GelMA fibers with or without agrin treatment (scale bars: 50 *μ*m). Reprinted with permission from Ebrahimi *et al.*, J. Tissue Eng. Regen. Med. **12**(11), 2151–2163 (2018). Copyright 2018 Wiley-VCH.

In another study, Kurashina *et al.* developed a technique to create complex organs using small tissue units [Figs. 9(a)–9(f)]. The units were capable of being assembled into parallel and reeled tissues. The tissue units consisted of microfibers like hepatic tissue units composed of co-cultured Hep-G2 cells and HUVECs. The co-culture conditions were optimized by changing the thickness of the core and the cell ratio.^181^ Using a PDMS guide, Kato-Negishi *et al.* developed neural tissue units by creating fiber-like neural tissues covered with a calcium alginate hydrogel layer. The units could be connected from both ends. The proposed technique was used to construct complex neural tissues and maintain the constructed tissue structures for around two weeks of culture.^182^

To create fiber-like cellular constructs, cell lines can be cultured on the surface of the biocompatible micro-/nano-fibers with great hypoallergenic and cell guidance made of different materials such as alginate, collagen, and GelMA. The fiber materials and their surface functionalization significantly influence cell bioactivity and behavior, including growth, proliferation, differentiation, and migration. For instance, micro-/nano-fibers with lobes and groves can enhance the cell adhesion and alignment along the fiber's lobes and grooves. This feature can be beneficial for the formation of neural pathways on a chip. In addition, culturing myoblasts on the grooved GelMA hydrogel fibers has been employed for the regeneration of muscle tissues [Figs. 9(g)–9(l)].^183^ Beyond this, the surface functionalization of the scaffolds by adhesive proteins such as collagen or fibronectin could improve cell proliferation, adhesion, and viability.

### D. Drug delivery

In the realm of pharmaceutics and disease treatment, the controlled release and targeted administration of medicines have long been the focus of interest. Also, simply raising the drug dose may result in negative responses and severe side effects in patients. As a result, innovative drug delivery methods must be developed so that medicines can be delivered in a controlled manner.^184^ Recently, microfluidic spun fibers have been extensively employed for drug delivery applications. They are typically fabricated out of hydrogel-based materials with a high capacity for the loading and delivery of macromolecules or drugs as well as their controlled release behavior, which depends on both the drug diffusion and fiber degradation rate.^38^

For instance, Ahn *et al.* encapsulated the ampicillin in microfluidic spun alginate fibers using a low-polarity isopropyl alcohol sheath flow.^185^ They have demonstrated that the isopropyl alcohol could cross-link the ampicillin and alginate fibers to significantly increase the loading capacity of the fibers. Due to the solvent exchange-induced phase separation and shear pressures inside the microchannel, the alginate chains align along the longitudinal direction and tightly compact together, contributing to the slow release of the ampicillin. The fibers were later employed for wound healing applications.^185^ Cui *et al.* also fabricated a dual-drug delivery system by encapsulating peptide hydrogel into alginate microfibers to improve the healing process of infectious wounds.^186^ Short peptides with a rapid self-assembling ability in the weak acidic solution were loaded with antibiotics before employing the recombinant bovine basic fibroblast growth factor (FGF-2)-alginate fiber encapsulation. The device had strong mechanical properties in which antibiotics were released faster than the growth factor from the peptide hydrogel. The developed dual-drug delivery system exhibited high antibacterial activity and improved the wound healing properties, according to both the *in vitro* and *in vivo* studies.^186^

Combining the droplet-based and continuous-flow microfluidics, He *et al.* also manufactured chitosan microfibers with a peapod-like structure to encapsulate water- and oil-soluble molecules for drug delivery applications.^74^ The use of chitosan combined with microfluidics has shown promising potential in developing targeted drugs with controlled release. The structure of fibers was adjustable by only varying the flow rates in the microfluidic system, a simple approach to develop a matrix for multi-compartment fibers. In another study, Aftab *et al.* showed a successful encapsulation of *Calotropis procera* extract (CpE) into microfluidic spun chitosan microfibers with an average efficiency of 77.125% ± 6.9%.^187^ To fabricate the fibers, they functionalized the chitosan with silver nanoparticles to improve the thermal stability and bioavailability of the drugs in the fibers. CpE was found to be effective in the treatment of human breast cancer, and this study proved that the developed system could be employed to suppress the proliferation of breast cancer cells (MCF-7) in humans.^188^ Microfluidic spun chitosan-based nanoparticles were also synthesized by Shamsi *et al.* for the transdermal dual-drug delivery applications.^189^ Nanoparticles blend was fabricated based on the poly(*N*-isopropylacrylamide-co-acrylic acid) and cellulose laurate to functionalize the chitosan fibers. They have shown that the developed nanoparticles-chitosan fiber system can be employed for the delivery of both the tretinoin and clindamycin phosphate in a controlled manner with minimum inhibitory and bactericidal concentrations.^189^ Finally, Zhang *et al.* synthesized alginate-chitosan microfluidic spun fibers for the prolonged and controlled ampicillin release in drug delivery applications.^190^ It was exhibited that the drug loading capacity and degradability time-scale of the developed drug delivery system can be easily controlled by regulating the concentration of chitosan and isopropyl alcohol as a low polarity sheath flow.^190^

### E. Optical sensors

Microfluidic technologies have also been employed to manufacture fluorescent spun fibers, which can be used as a matrix to fabricate optical fiber-based sensors due to their unique advantages, such as simple operation, rapid response, and high sensitivity and specificity. The structures of these fluorescence fibers are simple, but the material characteristics and fabrication processes of the fibers are significant to the sensing performances, making their design quite challenging.

**FIG. 10.**
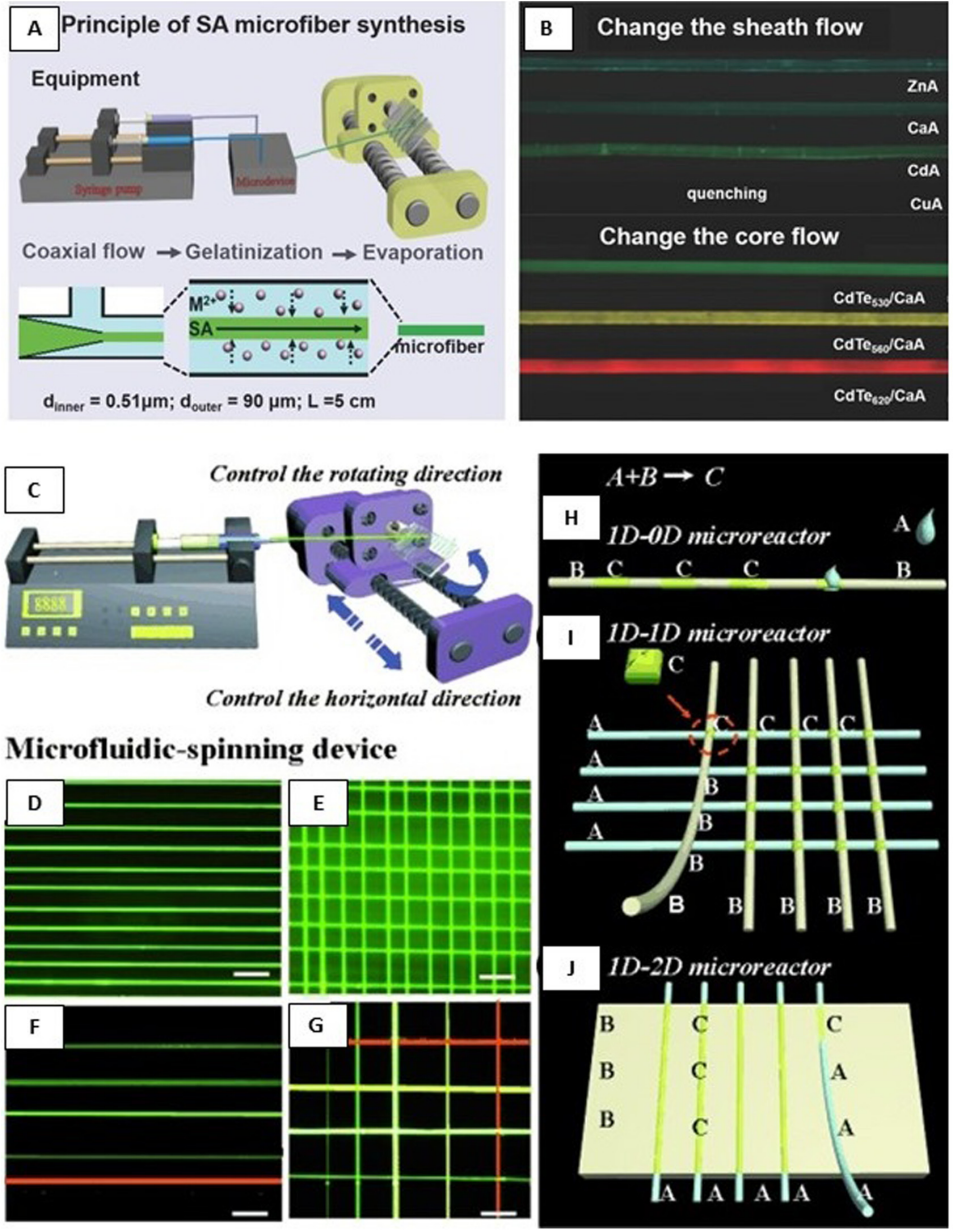
(a) Schematic illustrations of the microfluidic spinning equipment for the fabrication of M-alginate hydrogel microfibers and (b) fluorescent images of pure M-alginate microfibers and CdTe NCs (λem = 530, 560, and 620 nm) loaded Ca-alginate microfibers. Reprinted with permission from Zhang *et al*., Adv. Funct. Mater. **25**(47), 7253–7262 (2015). Copyright 2014 Wiley-VCH Publishing. (c) Schematic illustration of the microfluidic spinning device to fabricate microfibers suitable for optical sensor applications. [(d) and (f)] Fluorescence microscope images of parallel microarrays, and [(e) and (g)] orthogonal grids doped with a si1ngle fluorescent dye [(d) and (e)] and diverse fluorescent dyes [(f) and (g)]. Scale bars: 100 *μ*m. (h) 1D-0D, (i) 1D-1D, and (j) 1D-2D multidimensional microreactor arrays*.* Reprinted with permission from Xu *et al*., Angew. Chem., Int. Ed. **53**(15), 3988–3992 (2014). Copyright 2014 Wiley-VCH Publishing.

In a study, Cui *et al.* manufactured a fluorescent fibrous film using quantum dots (QDs) in a Y-shaped microchip.^191^ Quantum dots are semiconductor nanoparticles that glow a particular color when exposed to light. They have shown that QD-fluorescent microfibers are generated upon meeting Cd^2+^ and Se^2−^ ions at the knot of a Y-shaped microchip with great transparency and high optical characteristics, as well as outstanding flexibility and mechanical properties. The developed CdSe QDs microfibers were then milled into phosphor powders to fabricate the white light-emitting diode.^191^ Anisotropic fluorescent hybrid microfibers with distinct optical properties and delicate architectures have also been introduced by Zhang *et al*.^192^ They have functionalized hydrogel microfibers with Cd nanocrystals and employed them as optical labels for the multiplexed analytical assays. For instance, they have shown that photoluminosity of the microfibers can be quenched entirely and recovered in the presence of Cu^2+^ and Pb^2+^, respectively. The selective response toward different metal ions suggests that the hybrid fluorescent microfibers could be suitable for fabricating optical probes [Figs. 10(a) and 10(b)].^192^

Furthermore, Cheng *et al.* showed how amphiphilic QDs such as CdSe/ZnS QDs can be modified with beta-cyclodextrin (β-CD) and employed as color conversion materials to create a liquid crystal display backlight with a wide color range up to 112%.^193^ The amphiphilic QDs can also be easily integrated into polymers using microfluidic spinning technology to create fluorescent textiles suitable for flexible optical systems. They further introduced a functional platform with the ability to realize amphiphilic QDs for high-color-quality light-emitting applications.

Finally, Xu *et al*. utilized the microfluidic spinning method to fabricate fluorescent-dye-doped PVP microfibers and, thus, created multi-colored patterns of parallel arrays and grids [Figs. 10(c)–10(j)].^194^ The formation of hybrid polymer microarrays with fluorescent patterns by the use of intersection microreactors may offer a new avenue for the construction of ordered multidimensional configurations for various applications, such as optical microsensor arrays and high-quality display devices.^194^

## V. LIMITATIONS, FUTURE PERSPECTIVES, AND CONCLUDING REMARKS

This review presents an overview of the development of newly emerged microfluidic technologies for fabricating fiber-like structures on a micro-scale, leveraging different fabrication techniques, covering various types of materials, and enabling different fiber shapes and applications. There is a large body of literature focused on the use of microfluidic-based methods to produce cross-linkable microfibers for a specific application, while the importance of the fabrication technique has often been secondary to the materials and cross-linking mechanisms for enabling the targeted application. Thus, the current review discusses different microfluidic techniques employed in the literature for the fabrication of micro-/nano-fibers and highlights the importance of implementing these techniques for the effective and controlled production of fiber-like structures for specific applications. We summarize and classify different microfluidic platforms, including microfluidic chips, glass capillaries, or 3D printed devices, made of various materials such as a variety of polymers, glass, Teflon, and metals. We also highlight different mechanisms for the fiber fabrication using the microfluidic platforms, including hydrodynamic focusing, core–sheath flows, no-sheath flows, and co-flowing. Besides the fabrication methods, the diversity of applied materials and cross-linking mechanisms for the formation and solidification of the microfibers are also discussed here, in addition to the morphologies and characteristics of the obtained microfibers. Finally, emerging applications for the microfibers formed via microfluidic approaches are presented, such as micro-nano-based sensors, wearable electronics, tissue engineering, drug delivery, and optical sensors.

These applications are empowered due to the superior control over the fluid handling, flexibility of the fabrication techniques, good mechanical properties of the obtained microfibers, the possibility of manipulating multiphase fluid flows in a well-designed microchannel, and excellent tunability of fibers morphology and shape; the features enabled by the microfluidic technology. Owing to the laminar flow conditions that are typical of microfluidic systems, controlled mixing of fluids, well-controlled supply of flows, and tunable orientation of the formed structures are possible within simple microfluidic devices. From the perspective of fiber formation, microfluidic approaches are highly advantageous for fabricating fibers with controlled mechanical and physical properties as the fluidic microchannels incorporated in microfluidic devices allow the precise alignment of micro-/nano-fibers by the shearing flows. It is evident that microfluidic technologies provide a robust, versatile platform for the fabrication of micro-/nano-fibers, enabling researchers in various fields to take advantage of the wide range of morphologies, characteristics, and properties of the formed fibers. Despite the recent progress in this field, future development and research should focus on expanding the diversity of the microfluidic setup designs, strategies employed for fiber production, and processes for controlling fiber shape, size, and morphology. This development will pave the way for researchers to use a wider range of raw materials for fiber production and produce fiber-like structures with a variety of functionalities, which in turn will enable the application of the produced fibers for various emerging practical and even industrial applications.

Moreover, although remarkable research progress has been achieved in using microfluidic approaches for the fabrication of fiber-like structures, several challenges still need to be addressed to broaden the applicability of these approaches. Primarily, more microfluidic techniques for the spinning and fabrication of fibers need to be developed, accounting for different device designs, hydrodynamics technologies, and formation mechanisms to realize the manipulation of fiber morphology and properties. Furthermore, a vast majority of research efforts using microfluidic technologies focus on the use of alginate as a material for fiber production, while alginate suffers from several drawbacks. Put simply, due to the ionic cross-linking networks, alginate gels typically have inferior mechanical properties such as being brittle and unstretchable, lacking cell adhesion sites on the surface, preventing the encapsulated cells from mitigation and spreading, and exhibiting limited long-term stability in physiological conditions. Thus, the formation of microfibers made of alternative materials with enhanced performance and the respective enhancing strategies and cross-linking mechanisms should be further explored. In addition, although microfluidic techniques have shown great promise in producing biomimetic fibers, e.g., silk fibers, the mechanical properties of the regenerated fibers are still far from being comparable with the natural ones. To this end, more diverse microfluidic devices could be developed to produce microfibers with enhanced performance and improved strength. This is expected to be achieved by reducing the microfluidic device dimensions or increasing the shear effect, which will, in turn, lead to the reduction of the size of the fiber toward the nanoscale. Lastly, although many different types of microfluidic platforms ranging from 2D to 3D have been developed for the continuous production and collection of microfibers, the industrialized mass production of fibers using microfluidic technologies is still in its infancy. The low speed of fiber spinning is a significant drawback for the mass production of fibers through microfluidics. This narrowness in industrialized large-scale production of microfibers is predominantly on account of the complexity of the microfluidic device design and manufacturing and the highly precise conditions required for their operation. As such, further research on meaningful upscaling of the chip design for fabricating fibers using microfluidic technologies is deemed necessary, which is expected to ease the translation of microfluidic-based production of microfibers within the industrial and engineering facilities. Such progress will make the use of microfluidic methods to produce fiber-like structures more accessible not only for academic research but also for different applications in the industrial sector.

In conclusion, we believe that microfluidic approaches offer a powerful method for the controlled fabrication of various advanced microfibers made of different materials yielding multicomponent heterogeneous structures. These microfibers exhibit increasingly tunable shape, diversified morphology, and smaller fiber size, which will enable the fabrication of structures with different functionalities and complex surface geometries. Finally, the great possibility of producing microfibers with the desired properties and controlled performance provided by microfluidic technologies not only allows researchers to employ these methods for academic and fundamental applications such as various biomedical and pharmaceutical applications, but also opens the door to the possible upscale production of microfibers and their use in industry sectors such as the textile and food industry.

## Data Availability

Data sharing is not applicable to this article as no new data were created or analyzed in this study.
